# A Density Functional Theory Study of the Physico-Chemical Properties of Alkali Metal Titanate Perovskites for Solar Cell Applications

**DOI:** 10.3390/molecules29143355

**Published:** 2024-07-17

**Authors:** Shirzad Jouybar, Leila Naji, Saeedeh Sarabadani Tafreshi, Nora H. de Leeuw

**Affiliations:** 1 Department of Chemistry, AmirKabir University of Technology, No. 350, Hafez Avenue, Valiasr Square, Tehran 1591634311, Iran; shdjbr@aut.ac.ir; 2 School of Chemistry, University of Leeds, Leeds LS2 9JT, UK; 3 Department of Earth Sciences, Utrecht University, 3584 CB Utrecht, The Netherlands

**Keywords:** density functional theory, alkali-based titanate perovskite oxides, polymer and perovskite solar cells, charge transport layers, additional absorber

## Abstract

The urgent need to shift from non-renewable to renewable energy sources has caused widespread interest in photovoltaic technologies that allow us to harness readily available and sustainable solar energy. In the past decade, polymer solar cells (PSCs) and perovskite solar cells (Per-SCs) have gained attention owing to their low price and easy fabrication process. Charge transport layers (CTLs), transparent conductive electrodes (TCEs), and metallic top electrodes are important constituents of PSCs and Per-SCs, which affect the efficiency and stability of these cells. Owing to the disadvantages of current materials, including instability and high cost, the development of alternative materials has attracted significant attention. Owing to their more flexible physical and chemical characteristics, ternary oxides are considered to be appealing alternatives, where ATiO_3_ materials—a class of ternary perovskite oxides—have demonstrated considerable potential for applications in solar cells. Here, we have employed calculations based on the density functional theory to study the structural, optoelectronic, and magnetic properties of ATiO_3_ (A=Li, Na, K, Rb, and Cs) in different crystallographic phases to determine their potential as PSCs and Per-SCs materials. We have also determined thermal and elastic properties to evaluate their mechanical and thermal stability. Our calculations have revealed that KTiO_3_ and RbTiO_3_ possess similar electronic properties as half-metallic materials, while LiTiO_3_ and CsTiO_3_ are metallic. Semiconductor behavior with a direct band gap of 2.77 eV was observed for NaTiO_3_, and calculations of the optical and electronic properties predicted that NaTiO_3_ is the most appropriate candidate to be employed as a charge transfer layer (CTL) and bottom transparent conducting electrode (TCE) in PSCs and Per-SCs, owing to its transparency and large bandgap, whereas NaTiO_3_ also provided superior elastic and thermal properties. Among the metallic and half-metallic ATiO_3_ compounds, CsTiO_3_ and KTiO_3_ exhibited the most appropriate features for the top electrode and additional absorbent in the active layer, respectively, to enhance the performance and stability of these cells.

## 1. Introduction

Harvesting energy from cheap, plentiful, and natural sources using high-efficiency, low-cost devices remains the goal of many studies. In recent decades, research into photovoltaic (PV) technology has witnessed significant progress, primarily fueled by the increasing focus on renewable energy sources, employing materials that are not only non-toxic but also abundant [1,2,3,4]. Crystalline silicon remains the industry standard in PV devices, despite the high processing energies and, hence, costs. Cheaper PV devices are required to advance the field, especially for use in large-scale power production, but also in smaller, more portable, and distant applications, which has spurred a search for more readily available materials to substitute for silicon [5,6,7].

Emerging semiconductor-based solar cells, such as perovskite solar cells (Per-SCs) and polymer solar cells (PSCs), have attracted considerable attention in recent years. These solar cells typically feature a light-absorbent active layer located between a bottom transparent conductive electrode (TCE) and a top metal electrode [8,9]. Other important components are charge transport layers (CTLs), which include electron transport layers (ETL) and hole transport layers (HTL), which are required to maximize charge carrier collecting, alleviate charge extraction, and modify energy level alignment [10,11,12]. Various materials, such as nickel oxide and zinc oxide, have been employed as CTLs in both PSCs and Per-SCs, yielding relatively successful outcomes [13,14,15]. Under light irradiation, photo-generated excitons within the active layer are separated into electrons and holes, which then move toward the CTL/active layer interfaces. The ETL extracts and transports electrons to the lower WF electrode (cathode), while the HTL performs the same function for holes, directing them to the higher WF electrode (anode) [16]. While the main function of the electrodes is to gather and transfer holes and electrons, they should also have additional relevant attributes. Efficient TCEs are crucial components of these solar cell architectures, which should have high optical transmittance, excellent electrical conductivity, low sheet resistance, suitable WF, and high mechanical and thermal stability and be affordable. Presently, indium tin oxide (ITO) and fluorine-doped tin oxide (FTO) are the predominant TCEs employed in solar cells [17]. Despite notable progress having been made in enhancing the efficiency and stability of PSCs and Per-SCs, significant challenges hinder their commercialization [18]. Metal oxides have emerged as promising candidates for CTLs and TCEs owing to their superior chemical and thermal stabilities, suitable dielectric constants, and excellent charge mobility. These materials have been extensively studied and utilized in various capacities within PSCs and Per-SCs, including as CTLs, TCEs, buffer layers, and absorbent materials in the active layer. Moreover, they can be synthesized from inexpensive precursors, most of which involve low-temperature production [10,19,20,21]. Metal oxides for photovoltaic devices can align with the active layer, be optically transparent, and conduct electricity effectively. However, their constant features restrict their versatility among active layer materials, making many solar cell applications inflexible. One possible solution to these limitations is the incorporation of ternary metal oxides, which have band structures that may be adjusted by changing their chemical formula [22,23,24,25].

Perovskite-type oxides, as a class of ternary metal oxides, have garnered significant interest due to their simple structure and versatile applications. These compounds exhibit a wide range of electrical characteristics, from insulating to semiconducting to metallic, making them promising candidates for numerous technological applications, including solar cells [26,27]. Perovskites are often represented by the generic formula ABO_3_, where A and B are cations of varying atomic sizes. The alkali or earth-alkaline metals (A) are larger cations, whereas the transition metals (B) are smaller [26]. Perovskite oxides exhibit a variety of physical characteristics, including ferroelectricity, dielectricity, and piezoelectricity, which are primarily attributed to their capability to be chemically adjusted, allowing for small modifications in composition or crystal structure. This compositional tuning allows for the creation of various customized compounds with specific properties, such as optical transparency, conductivity, or catalytic activity, by substituting A and/or B-site cations in the ABO_3_ perovskite lattice [28]. Perovskite oxides with wide band gaps exceeding 2 eV have been utilized as CTLs and TCE in solar cells [29,30,31,32,33,34,35], whereas metallic compounds are widely utilized in PSCs and Per-SCs, for example, as absorbents in the active layer [36,37,38,39,40]. Research has also indicated that the utilization of magnetic materials as absorbents in the active layer of solar cells could potentially enhance their performance [41].

Extensive theoretical and empirical investigations have explored a broad spectrum of isomorphs of titanate-based perovskite oxides, encompassing configurations such as rhombohedral, orthorhombic, tetragonal, and cubic structures [42,43,44,45]. First-principle calculations have been employed to determine the properties and structures of these compounds, such as their electronic band structure and ferroelectricity, as well as their optical characteristics, including absorption, emission, transmission, and reflection [46,47,48].

Flexible solar panels require lightweight materials, mechanical flexibility, and the capacity to be molded into complex shapes, such as roof panels for electric vehicles, folding umbrellas, and camping tents [49]. Thin-film polymer and perovskite solar cells are lightweight and mechanically flexible, which makes them suitable for flexible substrates, including the bottom electrode, charge-transporting layers, the active layer, and the top electrode in planar flexible solar cells, where they should be able to tolerate repeated bending processes [50]. However, only a limited number of works have reported the mechanical and thermoelectric properties of the titanate-based perovskite oxides studied in this work.

In the present work, we have employed calculations based on the DFT within the generalized gradient approximation (GGA-DFT) to determine the structural, electronic, optical, magnetic, thermal, and elastic properties of a series of alkali-based ATiO_3_ in their most stable structures. The elastic properties were evaluated to assess the flexibility and thermal stability of these materials for use in PSCs and Per-SCs structures to enhance their performance and lifetime. We also evaluated the potential of any of these perovskite-type oxides for the CTLs/TCE, top electrode, or absorbent in PSCs and Per-SCs. Prior research on titanates with the perovskite structure has mainly concentrated on a few specific alkaline earth-based systems, i.e., CaTiO_3_, SrTiO_3_, and BaTiO_3_ [51,52,53,54,55,56]. While individual properties of alkali metal-based titanate perovskite oxides (ATiO_3_, A=Li, Na, K, Rb, Cs) have been studied in prior research, our work uniquely contributes by systematically examining their critical properties within a single study. This comprehensive approach provides significant insights into the applicability of these materials for solar cell technologies, where our results indicate that certain ATiO_3_ compounds are promising candidates to integrate into PSCs and Per-SCs cells, where they could contribute to enhanced performance, thermal stability, and mechanical flexibility.

## 2. Methodology

In this work, we have employed the Vienna Ab initio Simulation Package (VASP) [57,58] for the DFT calculations. The Perdew–Burke–Ernzerhof (PBE) functional for the generalized gradient approximation (GGA) [59] was used to determine the exchange and correlation energies within the projector augmented wave (PAW) method [60,61]. We have employed the DFT-D3 method by Grimme to include the long-range Van der Waals (vdW) forces to improve the energy description of the system [62,63].

The electron wave functions are expanded using plane waves as basis sets with a cut-off energy of 600 eV. The convergence criteria for the Hellmann–Feynmann forces and tolerance limit for the energy during structure optimization are set to 0.01 eV/Å and 10^−5^ eV, respectively. While we have used an 11 × 11 × 11 Monkhorst Pack of k-point grides to sample the Brillouin zone in the energy minimizations, the size of the mesh grid in the k-space during the calculations of the electronic properties was set at 21 × 21 × 21. 

It is a well-known limitation of GGA-DFT calculations, as used in this study, that they tend to underestimate the electronic structure and band gaps of materials. However, here we have focused on a comparative analysis of the alkali-based titanate perovskite oxides, and since the same DFT-GGA approach was applied consistently across all compounds, the relative comparisons and trends observed remain valid. The systematic underestimation affects all compounds in a similar manner, thus allowing for a meaningful relative assessment of their properties. In the future, more accurate methods such as hybrid functional calculations, which incorporate a portion of exact exchange from the Hartree–Fock theory, could be employed. These methods have been shown to provide a better estimation of the electronic properties, including band gaps, and we plan to incorporate hybrid functional methods in future work where we will focus in more detail on the most promising candidates from the current study.

The elastic coefficients were calculated by generating 7 distorted structures for each strain pattern, including three positives and three negatives under the maximum strain amplitude of 0.015, where the structures are optimized with convergence criteria of total energy within 1 × 10^−8^ eV/atom, the ionic Hellmann–Feynman forces within 1 × 10^−2^ eV/Å, and maximum ionic displacement within 1 × 10^−4^ Å.

Each crystal structure with a certain symmetry has different numbers of independent elastic constants. The inverse of each stiffness tensor, $c_{ij}$, is the compliance tensor, $s_{ij}={c_{ij}}^{-1}$. The crystal bulk modulus (B), shear modulus $\left(G\right),$ Young’s modulus (E), and Poisson’s ratio $\left(\nu \right)$ were calculated according to the Voigt, Reuss, and Hill approximations [64,65,66]. According to the Hill approximation [64], the bulk modulus $\left(B_{H}\right)$ and shear modulus ${(G}_{H})$ are calculated according to Equations (1) and (2) as:

(1) $$B_{H}=\frac{1}{2}\left(B_{V}+B_{R}\right)$$

(2) $$G_{H}=\frac{1}{2}\left(G_{V}+G_{R}\right)$$

where the two parameters of the bulk modulus and shear modulus, according to the Voigt and Reuss approximations [65,66] $B_{V}{,B}_{R},\;G_{V},$ and $G_{R}$, are averaged by employing Equations (S1) to (S4).

Young’s modulus $\left(E_{H}\right)$ and Poisson’s ratio ($v_{H}$) are calculated using the bulk modulus ${(B}_{H})$ and shear modulus ($G_{H}$) from the Hill scheme, using Equations (S5) and (S6).

Using the calculated $E_{H}$ and $v_{H}$ and crystal structure parameters, the theoretical minimum thermal conductivity ($\kappa_{min}$) was obtained as the lowest limit of the thermal conductivity value [67,68,69,70,71,72]. According to the Clarke model [73], the theoretical minimum thermal conductivity can be calculated via Equation (3):

(3) $$\kappa_{min}=0.87k_{B}{\left.\left(\frac{M}{n\rho N_{A}}\right.\right)}^{-2/3}\sqrt{\frac{E}{\rho }}$$

where *k_B_* refers to the Boltzmann constant, *E* is Young’s modulus, *ρ* is the density of each perovskite crystal, *N_A_* is Avogadro’s number, *M* is the molecular weight, and *n* is the number of atoms. In this study, we have used the modification by Liu et al. [67,68,69] to the Clarke model through Equation (S7), which was proposed for the calculation of the minimum thermal conductivity from DFT calculations of the elastic parameters (h is Plank’s constant).

Another critical constant of thermal properties, the Debye temperature, is derived from Equation (4): [74]

(4) $$\theta_{D}=\frac{h}{k_{B}}{\left[\frac{3n}{4\pi }\left(\frac{N_{A}\rho }{M}\right)\right]}^{1/3}\nu_{m}$$

where $\nu_{m}$, the average sound velocity, is calculated using Equation (5) [74]:

(5) $$\nu_{m}={\left\{\frac{1}{3}\left[2{\left(\frac{1}{2+2\nu_{H}}\right)}^{-3/2}+{\left(\frac{1}{3-6\nu_{H}}+\frac{2}{3+3\nu_{H}}\right)}^{-3/2}\right]\right\}}^{-1/3}\sqrt{\frac{E_{H}}{\rho }}$$

The elastic and thermal properties were calculated using VASPKIT [75], and the spatial dependence figures of the elastic properties were visualized by ELATE [76].

## 3. Results

### 3.1. Structural Properties

**Figure 1:**
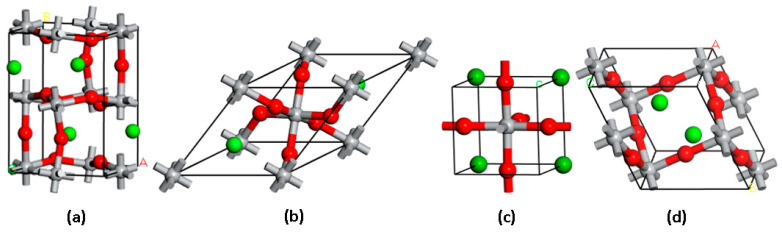
Crystal structures of the studied perovskite oxides with (**a**) orthorhombic A_4_Ti_4_O_12_ (A=Li), (**b**) tetragonal ATiO_3_ (A=K, Cs), (**c**) trigonal A_2_Ti_2_O_6_ (A=Rb), and (**d**) monoclinic A_2_Ti_2_O_6_ (A=Na) lattices. Atoms of A, O, and Ti are shown as green, red, and grey spheres, respectively.

Figure 1 illustrates the crystal structures of the investigated perovskite oxides. The Open Quantum Materials Database (OQMD) was used to obtain the structures, which were determined to be the most stable based on the estimated formation energies [77,78]. Our investigation focused on perovskite oxides characterized by the general formula ATiO_3_, where A represents the alkaline metals Li, Na, K, Rb, and Cs. These compounds are found in different crystal lattice structures, including orthorhombic, tetragonal, trigonal, and monoclinic phases. Specifically, the unit cells are (a) orthorhombic A_4_Ti_4_O_12_ (A=Li), (b) tetragonal ATiO_3_ (A=K, Cs), (c) trigonal A_2_Ti_2_O_6_ (A=Rb), and (d) monoclinic A_2_Ti_2_O_6_ (A=Na) crystal lattices. Table 1 provides comprehensive information on the structural characteristics and formation energies of the perovskites under investigation.

Based on the Goldschmidt approach, the tolerance factor (t-factor) is the most widely used and effective method to estimate the stability of perovskite oxide structures [79]:

(6) $$t=\frac{\left\langle r_{A}\right\rangle +\left\langle r_{O}\right\rangle }{\sqrt{2}\left(\left\langle r_{B}\right\rangle +\left\langle r_{O}\right\rangle \right)}$$

The mean ionic radii of the A, B, and O atoms are represented by $\left(r_{A}),(r_{B}\right)$, and ($r_{O}$), respectively. The t-factor indicates if A cations could fill the cubic structure corners by penetrating the spaces between the octahedral structures [80]. When the t-factor shifts from 0.8 to 1.1, the perovskite structure becomes feasible, while maintaining structural stability. Within the lower end of this range, the BO_6_ octahedra may undergo tilting, and symmetry reduction is possible, albeit with potential distortion. However, as the t-factor exceeds 1.1, it suggests that the A site cation is overly large, typically impeding the perovskite formation. Conversely, a t-factor below 0.8 indicates that the A site cation is too small, often resulting in alternative structural arrangements [81]. According to Table 1, the t-factor falls within the range of 0.85 to 1.04 for the examined compounds, confirming the stability of their respective structures.

### 3.2. Electronic and Magnetic Properties

The efficiency of both Per-SCs and PSCs is directly affected by the bandgap and energy levels of the HTL and ETL. In order to be suitable as CTLs, perovskite oxides need to possess a wide bandgap [31,33]. This characteristic enables more light to penetrate and reach the photosensitive active layer, enhancing the overall efficiency of the device [82]. The bandgap is the energy difference between the highest energy level of the valence band (VBM) and the lowest energy level of the conduction band (CBM) [25]. The electronic structure and inherent properties of a number of perovskite oxides, such as energy levels, bandgap, band structure, Fermi energy, and density of states, have been estimated by DFT calculations [83,84,85]. Here, we have used spin-polarized calculations to provide a comprehensive assessment of the electronic structures of the range of investigated perovskites, characterize their magnetic properties, and evaluate their capacity to be applied in PSCs and Per-SCs. Since ferromagnetic materials have shown significant potential to absorb a meaningful amount of visible light, their integration into solar cells may improve the efficiency of these devices [41].

**Figure 2:**
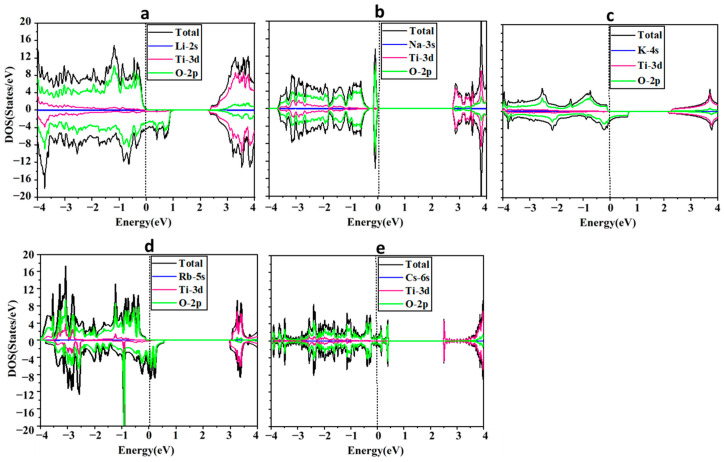
Total and partial density of states for (**a**) LiTiO_3_, (**b**) NaTiO_3_, (**c**) KTiO_3_, (**d**) RbTiO_3_, and (**e**) CsTiO_3_. The Fermi level is set to zero.

We have analyzed the band structure (BS) and density of states (DOS) of the ATiO_3_ (A=Li, Na, K, Rb, and Cs) perovskites. Figure 2a–e shows the spin-polarized total and partial density of states (PDOS) distributions for all the considered ATiO_3_ systems obtained using the photon energy from −4 to 4 eV. Our calculations show that ATiO_3_ (A=Li, K, Rb) are ferromagnetic, while ATiO_3_ (A=Na, Cs) are non-magnetic structures. The total spin magnetic moments per formula unit of each of these compounds and their partial spin magnetic moments per atom are summarized in Table 2.

Table 3 provides a summary of the energy levels of the VBM and CBM, along with the energy gap around the Fermi level, set at zero eV for all structures. Based on the location of the Fermi level, which crosses the energy levels of each structure in both spin-up and spin-down channels, we have identified the electronic nature of each structure, i.e., metallic, half-metallic, or semiconductor. Further insights can be obtained from the direct or indirect nature of the bandgap in the semiconductor compound NaTiO_3_. Our findings reveal that in this material, electrons and holes recombine at the single symmetry point (L_2) of the Brillouin zone, indicating that this compound has a direct bandgap.

#### Electronic and Magnetic Properties of ATiO_3_ (A=Li, Na, K, Rb, and Cs)

Figure 2a–e shows the total DOS of the alkali metal-based titanate perovskite structures. Figure 2a,c,d shows that three ATiO_3_ structures (A=Li, K, and Rb) are asymmetric in spin-up and spin-down directions, suggesting magnetic moments of 0.836, 0.781, and 0.820 µB per formula unit, respectively, with O-2p as the main source of the magnetization. However, the symmetric distributions of spin-up and spin-down for NaTiO_3_ (Figure 2b) and CsTiO_3_ (Figure 2e) resulted in zero net magnetic moments (see Table 2). Figure 2c,d and Table 2 reveal that KTiO_3_ and RbTiO_3_ possess similar electronic properties as half-metallic ferromagnetic materials. Spin-down channels have metallic behavior due to overlapping valence bands across the Fermi level at zero energy. In contrast, spin-up channels display semi-conductor behavior with wide band gaps of 2.333 and 3.177 eV, respectively. Half-metallicity has already been reported for RbTiO_3_ [86], as well as other perovskite oxides [87,88,89,90], double perovskites [91,92,93], perovskite compounds [94,95], and metal oxides [96,97]. Ferromagnetic materials have yet to be used in the fabrication of solar cells, but they could be a major component in developing solar cell technology in the future. Due to spin-dependent transition selection criteria, ferromagnetic materials may have longer lifetimes in excited states [41], which could affect the low conversion efficiency of solar cells. Long lifetimes of excited states are critical for photovoltaic energy conversion systems because they provide more time for photo-generated carriers to be collected before recombination, thereby enhancing the efficiency of the cells. Moreover, ferromagnetic materials have shown the potential to adsorb a considerable amount of visible light [41]. Therefore, KTiO_3_ and RbTiO_3_, as half-metallic structures, and LiTiO_3_ with metallic characteristics, which all exhibit ferromagnetism, can be used as additional absorbent materials in the active layer of PSCs and Per-SCs to promote their efficiency.

LiTiO_3_ (Figure 2a) and CsTiO_3_ (Figure 2e) are metallic, as the Fermi level crosses the energy states in spin-up and spin-down directions (see Table 3). The electronic properties of CsTiO_3_ are in agreement with results reported elsewhere [86]. In PVs, improvement in light harvesting is one of the most important factors to enhance the efficiency of solar cells. Enhanced photon harvesting could be accomplished by trapping light utilizing metallic nanoparticles at the interface or inside the active layer [98,99]. When metallic nanoparticles are exposed to light, the particles either absorb the light or scatter it, depending on their size. When light is absorbed by nanoparticles (size ˂ 20 nm), they act like sub-wavelength antennae because of localized surface plasmon resonance (LSPR) excitation. Particles larger than 20 nm serve as key elements for sub-wavelength scattering, which assists in capturing incoming light [36,38]. Moreover, as previously mentioned, metallic compounds are generally utilized as the top electrode in solar cells. Therefore, LiTiO_3_ and CsTiO_3_, which show metallic behavior, could be applied as the top electrode (cathode) or auxiliary absorbent in the active layer of PSCs and Per-SCs.

However, NaTiO_3_ is a semiconductor where the VBM is positioned slightly below the Fermi level (0 eV). This results in a bandgap of 2.771 eV between the VBM (−0.021 eV) and CBM (2.750 eV) in both the spin-up and spin-down channels, indicating that no energy state crosses the Fermi level (see Figure 2b and Table 3). A similar band structure with a close band gap has been reported for NaTiO_3_ in other studies [100], and as the only semiconductor with a wide bandgap, NaTiO_3_ could be considered a suitable candidate for CTL or TCE in PSCs and Per-SCs.

Figure 2 shows the PDOS to provide a more detailed understanding of the role of each orbital and localized energy state in the valence and conduction bands and their hybridization and contribution to the atomic levels within the perovskites. The results reveal that in all structures the O-2p states are located mainly in the valence band region with a small contribution from Ti-3d and A-s (A=Li, Na, K, Rb, and Cs) electrons in both spin-up and spin-down states. The half-metallic feature of KTiO_3_ and RbTiO_3_ and the metallic characteristics of LiTiO_3_ and CsTiO_3_ are largely due to O-2p states since they occupy the Fermi level in the spin-down channels in KTiO_3_ and RbTiO_3_, and both spin-up and spin-down channels in LiTiO_3_ and CsTiO_3_. The major peaks in the conduction bands are primarily due to Ti-3d with very few localized energy states of O-2p and A-s for both up- and down-spin channels.

**Figure 3:**
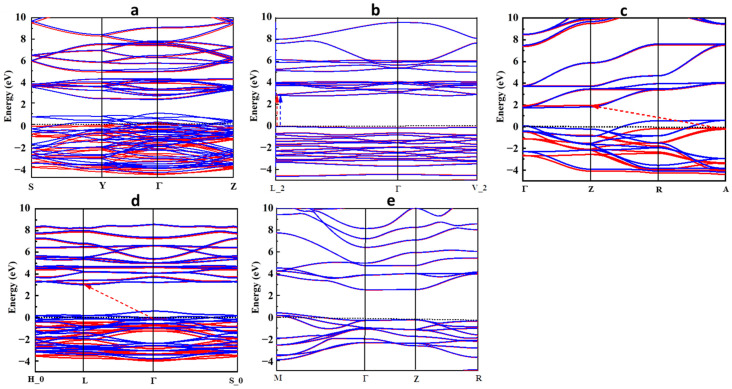
Band structure for (**a**) LiTiO_3_, (**b**) NaTiO_3_, (**c**) KTiO_3_, (**d**) RbTiO_3_, and (**e**) CsTiO_3_. Red and blue bands correspond to the spins up and down, respectively. The Fermi level is set to zero. The bandgaps of half-metallic and semiconductor structures are shown with red and blue dashed arrows in the spin-up and -down channels, respectively.

The spin-polarized band structures (BSs) of the perovskite oxide compounds, including alkali metals, are shown in Figure 3a–e. In LiTiO_3_ and CsTiO_3_, the BS plots in Figure 3a,e confirm the results of the DOS, indicating their metallic nature and asymmetric and symmetric distribution of spin-up and -down of the electronic states of LiTiO_3_ and CsTiO_3_, respectively. In NaTiO_3_ (Figure 3b), the highest edge of the VB and lowest edge of the CB appear at a single symmetry point (L_2) of the Brillouin zone with a direct band gap energy of 2.771 eV in both spin-up and spin-down directions, which is consistent with the DOS calculation. The BS results of KTiO_3_ and RbTiO_3_ in Figure 3c,d indicate indirect bandgaps of 2.333 and 3.177 eV in their spin-up channels from the symmetry points of A to Z and Γ to L, respectively, and metallic properties of these compounds in the spin-down directions, where the electronic band states cross the Fermi level.

### 3.3. Optical Properties

The optical characteristics of a material, such as absorption, transmission, reflection, and emission, provide crucial insights into its behavior under varying photon energies [2,101]. However, simulations focused on optimizing HTLs and ETLs in Per-SCs and PSCs are still lacking, despite the extensive experimental efforts dedicated to improving their performance and even though the electronic and optical properties of CTLs are crucial to the effective functioning of these devices [102,103].

The dielectric constants (ε(ω)), comprising a real part ($\varepsilon_{1}(\omega )$) and an imaginary part ($\varepsilon_{2}(\omega )$), play a critical role in determining the optical properties of a material. In addition, other critical optical characteristics comprise the refractive index (n(ω)), extinction coefficient (k(ω)), reflectivity (R(ω)), energy loss function (L(ω)), absorption coefficient (α(ω)), and optical conductivity (σ(ω)), where ω denotes the angular frequency of phonons [104].

Using appropriate equations, the subsequent sections will outline the intricate relations between the optical features and how these properties depend on each other. A number of optical features of titanate-based perovskite oxides have been researched in the past [54,86,100,105,106,107,108], but here we will provide a comprehensive analysis for completeness and comparison.

Our evaluation primarily focuses on the 1–4 eV spectral range, commonly referred to as the solar range, which encompasses the visible region. The relationship between photon energy (in eV) and wavelength (in nm) can be expressed inversely $E\left(eV\right)=\frac{1240}{\lambda (nm)}$. Consequently, the visible region spans approximately from 1.77 eV to 3.10 eV, corresponding to wavelengths ranging from approximately 400 nm to 700 nm [2].

#### 3.3.1. Dielectric Function

The dielectric function is an important characteristic directly linked to the rate of charge-carrier regeneration in materials utilized within solar cells. Its significance lies in providing insights into the operational characteristics and efficiency of optoelectronic devices, particularly in the context of photovoltaic applications. In PSCs and Per-SCs, recombination rates play a crucial role in determining device performance. Higher recombination rates lead to increased losses of photo-generated charge carriers, thereby compromising the overall efficiency of the solar cell. Modifying the dielectric constant of materials employed within these devices makes it possible to mitigate recombination rates and improve charge-carrier regeneration [109].

The dielectric function that relies on frequency consists of a real and an imaginary part, which are interconnected through the relationship of $\varepsilon \left(\omega \right)=\varepsilon_{1}\left(\omega \right)+{i\varepsilon }_{2}(\omega )$. The prediction of the material’s absorptivity can be achieved via the imaginary component, denoted as $\varepsilon_{2}\left(\omega \right)$, shown in Equation (7) [110]:

(7) $${i\varepsilon }_{2}\left(\omega \right)=\left(\frac{e^{2}\hbar^{2}}{\pi m^{2}\omega^{2}}\right)\sum_{\nu ,c}{\left|\left\langle \psi_{c}\left|e_{j}.\vec{P}\right|\psi_{\upsilon }\right\rangle \right|}^{2}\delta {(E}_{c}-E_{\upsilon }-\hbar \omega )$$

Here, e and m represent the charge and mass of an electron, respectively, $\vec{P}$ denotes the momentum operator, and $\hat{e_{j}}$ signifies the unit vector indicating the direction of the external electromagnetic field energy. In the context of the valence band, the valence energy is denoted by $E_{\upsilon }$, and the empty wave function by $\psi_{\upsilon }$, respectively. In contrast, the conduction energy and full-wave functions connected to the conduction band are denoted by $E_{c}$ and $\psi_{c}$, respectively.

When it comes to determining the dispersion and polarization properties of electromagnetic radiation inside a material, the real part $\varepsilon_{1}\left(\omega \right)$ is an extremely important factor that should be considered. Through the Kramers–Kronig connection, shown mathematically in Equation (8), it is connected to the imaginary section ($\varepsilon_{2}\left(\omega \right)$) [111], where P stands for the primary value of the integral.

(8) $$\varepsilon_{1}\left(\omega \right)=1+\left(\frac{2}{\pi }\right)P\int_{0}^{\infty }\frac{\overset{´}{\omega }\varepsilon_{2}\left(\overset{´}{\omega }\right)}{\overset{´}{\omega^{2}}-\omega^{2}}d\overset{´}{\omega }$$

**Figure 4:**
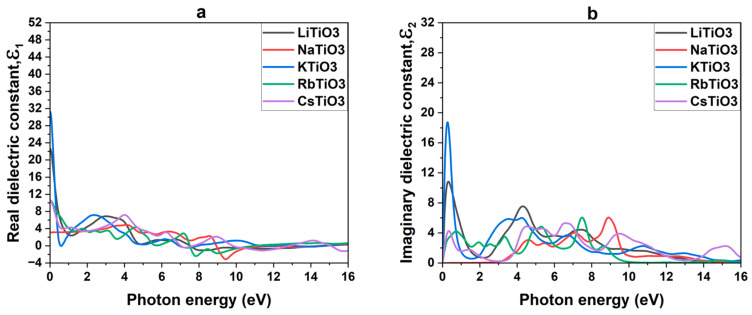
Real (**a**) and imaginary (**b**) dielectric constants ATiO_3_ (A=Li, Na, K, Rb, and Cs).

Figure 4a provides an illustration of the real dielectric constants $\varepsilon_{1}\left(\omega \right)$, and in Table 4, the static values of the dielectric constants at zero energy, denoted as $\varepsilon_{1}\left(0\right)$, are shown. It is evident that the dielectric constants decrease from zero energy in compounds containing alkali metals, with the exception of sodium, which exhibits the lowest value at zero energy and then displays a tiny rise thereafter. Negative values of $\varepsilon_{1}\left(\omega \right)$ arise after an additional increase in photon energy from the visible area to the ultraviolet (UV) region, which indicates that perovskite structures exhibit a significant reflection of incoming light and limited transmission through their surface in the UV region, as documented in prior research [86,112,113]. The evaluation of the studied compounds reveals a notably positive value of the real dielectric constant $\varepsilon_{1}\left(0\right)$ within the solar region. This characteristic suggests that these compounds exhibit less reflection from their surfaces in this spectral range, indicating favorable optical properties and transparency. In particular, NaTiO_3_ stands out among the studied structures due to its lowest static value of $\varepsilon_{1}\left(0\right)$, which remains consistently low and constant throughout the solar region. This distinctive feature positions NaTiO_3_ compounds as promising candidates for TCE and CTL in optoelectronic devices.

By examining the imaginary component of the dielectric function, we can gain valuable insights into the mechanisms governing light absorption in materials. For perovskite oxides to be viable candidates as CTLs or TCEs, it is crucial to engineer materials with minimal light absorption in the relevant spectral solar region, particularly in the visible range where solar irradiance is most abundant. This would enable efficient light transmission through the layers, facilitating optimal photon absorption within the active layer of the solar cell. Figure 4b illustrates the dynamics of the imaginary portion of the dielectric property, denoted as $\varepsilon_{2}\left(\omega \right)$, with energy amounts ranging from 0 to 16 eV for the studied substances.

As can be observed across all investigated structures, the positive values of $\varepsilon_{2}\left(0\right)$ and the presence of various peaks in this portion of the dielectric function indicates inter-band transitions of electrons from their filled valence band to the empty conduction band. This characteristic signifies the absorptive behavior of the compounds, a crucial aspect of their functionality in solar cells. As in preceding investigations, intra-band transitions were not considered in the materials under discussion. Although intra-band transitions could contribute to the overall optical behavior, the absorbance properties within the examined energy range are mostly determined by inter-band transitions [114,115]. A decline in absorption approaching the end of the UV spectrum, indicative of less interaction between the materials and incoming light at higher photon energies, is seen in all compounds, although this absorption is most pronounced in the center region of the UV area. Notably, for NaTiO_3_, the absorption remains nearly zero until a photon energy value of 3 eV (within the solar region). This characteristic renders NaTiO_3_ almost transparent in the solar region, making it well-suited for applications such as CTL and TCE in solar devices. Moreover, the imaginary component of the dielectric function indicates the non-zero absorption of some metallic and half-metallic compounds with ferromagnetic characteristics (Rb, K, and Li-based titanates) in the solar region, which confirms the promising features of these compounds as absorbents in the active layer.

#### 3.3.2. Refractive Index and Extinction Coefficient

The refractive index $n\left(\omega \right)$ and extinction coefficient $k\left(\omega \right)$ are quantities that help us understand the degree of transparency and capacity of absorbing incident light. The refractive index has been found to help analyze the behavior of incident light on a material, i.e., how much light is refracted after entering it [113,114]. The mentioned parameters, including n(ω) and k(ω), are determined by Equations (S8) and (S9).

**Figure 5:**
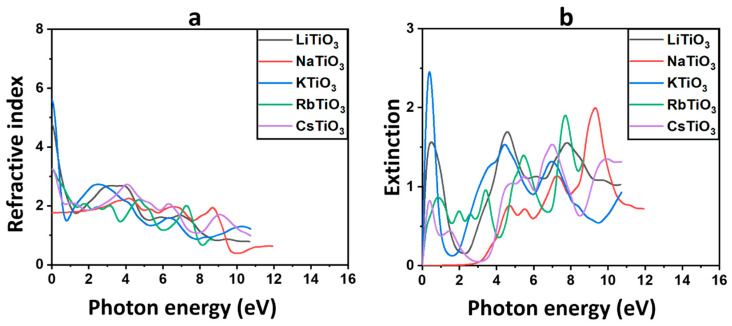
The refractive index and (**a**) and extinction coefficient (**b**) for ATiO_3_ (A=Li, Na, K, Rb, and Cs).

Figure 5a shows the spectrum of the predicted refractive indices as they change with the energy of the photon. Table 4 provides the static refractive indices $n\left(0\right)$ of the structures at zero energy. It is noteworthy that KTiO_3_ and LiTiO_3_ exhibit notably higher values compared to the other perovskite oxides examined in this study. Anticipated trends in variations of the real dielectric constant and the refractive index suggest a closely correlated relationship [112]. The observed slow rise in the refractive index of the compound containing Na, along with the abrupt decrease from zero energy in other alkali-based titanate structures except Na, align closely with the trends already noted for the dielectric constants. In addition, for all considered structures, the peaks lie in the UV region with values less than 3.0, where $n\left(\omega \right)$ is seen to decrease with increasing photon energy, indicating reduced interactions of incident light with the materials. Among all the investigated perovskite oxides, NaTiO_3_, characterized by semiconductor properties in its electronic structure, exhibits the lowest refractive indices within the solar region. This observation is in line with the behavior observed in $\varepsilon_{1}\left(\omega \right)$. The higher transparency of the material, indicated by the smaller value of $n\left(\omega \right)$, corresponds to its potential role as a TCE or CTL. Hence, considering the semiconductor nature of NaTiO_3_ and its highest transparency within the visible spectrum, it can be inferred that NaTiO_3_ holds promise as a suitable candidate for deployment as the bottom electrode and HTL or ETL in both normal and inverted architectures of Per-SCs and PSCs.

The shift of electrons from the VB to the CB in dielectric materials is reflected in the variation of k(ω) with photon energy. This relationship is depicted in Figure 5b [105]. For the Na-based structure, it is evident that k(ω) remains close to zero across the majority of the solar region, with peaks emerging throughout the UV region near 10 eV. This suggests that incident photons within the solar region below 4 eV are not significantly absorbed, while maximum light diffusion into the compound occurs in the UV region. The calculated k(ω) emphasizes that NaTiO_3_ is a promising CTL or TCE candidate for PSCs and Per-SCs. In contrast to the Na-based structure, there is a noticeable trend in the behavior of k(ω) among the Cs-, Rb-, K-, and Li-based titanates. These structures exhibit a sharp increase in k(ω) from zero energy, followed by a subsequent decline to lower energy values close to 2 eV. Subsequently, there is a rise again in the solar region before reaching 4 eV. The observed highest values of k(ω) in the solar region, particularly for LiTiO_3_ and KTiO_3_, indicate their significant absorption characteristics within this spectral range. Consequently, these structures are considered less suitable for application as CTLs or bottom electrodes in solar cells, as they lack the requisite transparency and instead absorb a substantial portion of incident light. Nevertheless, due to their absorbent properties within the solar region, compounds like KTiO_3_, characterized by ferromagnetic behavior, hold potential for application as absorbers in the active layer of PSCs and Per-SCs. The calculated refractive index $n\left(\omega \right)$ and extinction coefficient k(ω) of the pure material are in agreement with experimental results [116].

#### 3.3.3. Absorption Coefficient

**Figure 6:**
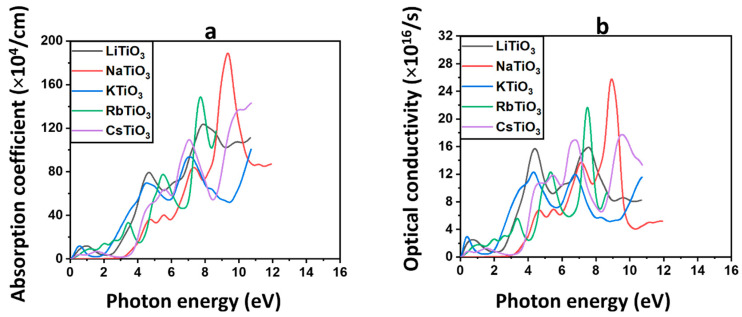
The absorption coefficient and (**a**) and optical conductivity (**b**) for ATiO_3_ (A=Li, Na, K, Rb, and Cs).

According to Equation (S10), the absorption coefficient $\alpha \left(\omega \right)$ is an indicator of the level at which a material has the capability to capture photons of light with an energy of ℏω [117]. Figure 6a shows the absorption spectra of all the examined materials. In the semiconductor structure of NaTiO_3_, negligible absorption or zero absorption is observed in the solar region, suggesting high transparency. This characteristic implies that most of the light within this region will pass through the material, rendering it a promising candidate for CTLs and TCE [112,118,119]. All other structures, including Cs, Rb, K, and Li, exhibit absorption in the solar region, rendering them unsuitable for applications requiring high transparency like CTLs. However, this observation underscores the potential role of the half-metallic structure of KTiO_3_ as an additional absorbent in the active layer of solar cells. As shown in Figure 6a, $\alpha \left(\omega \right)$ exhibits an upward rise with the energy of light in the UV region. The highest absorption values for each of the materials are listed in Table 4. Notably, NaTiO_3_ demonstrates the greatest absorption in the UV region, reaching 188.80 × 10^4^ cm^−1^, which corresponds to 9.34 eV. Consequently, NaTiO_3_ is regarded as the most absorptive material among all the studied perovskite oxides in the UV region of the electromagnetic spectrum. It is worth mentioning that the optical properties of these titanate-based perovskite oxides in PSCs and Per-SCs have not been evaluated in the UV region but rather in the solar region, as previously indicated.

#### 3.3.4. Optical Conductivity

The relationship between photon energy and the optical conductivity parameter, σ(ω), which represents the material’s conductivity as a result of photon-induced optical stimulation (ref. [120]), is obtained from Equation (S11) [121] and is shown in Figure 6b. The optical conductivity obeys analogous behavior with increasing energy as was seen in the absorption and extinction coefficient of the material. It is zero or negligible in most of the solar region for Na, indicating no optical interaction and excitation for this structure. Consistent with the optical characteristics mentioned earlier, the σ(ω) findings validate the possibility of using NaTiO_3_ as bottom TCE and CTL in PSCs and Per-SCs. Therefore, even the slightest incident light onto this material would pass through and reach the active layer in solar cells. However, the non-Na alkali metal materials demonstrate light interaction and optical excitation in the visible region, which prohibits their use as CTLs or TCEs. Additionally, the predicted light interaction in the solar region confirms the findings obtained from the assessment of absorption coefficients, indicating the potential of the ferromagnetic compound KTiO_3_ to serve as an additional absorbent in the active layer. The σ(ω) values are consistent with the $\alpha \left(\omega \right)$ and rise to higher values in the UV region. The maximum optical conductivities for all materials are listed in Table 4. NaTiO_3_, and RbTiO_3_ are the most conductive compounds in the UV region of the electromagnetic spectrum, possessing optical conductivities of 25.76 × 10^16^ and 21.66 × 10^16^ s^−1^, corresponding to 7.45 and 8.90 eV, respectively.

#### 3.3.5. Reflectivity

**Figure 7:**
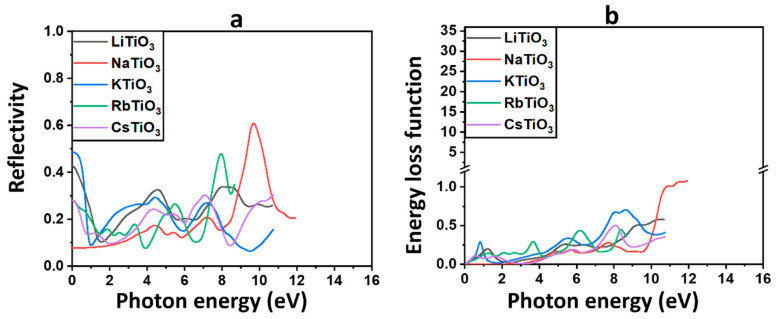
The reflectivity (**a**) and energy loss function (**b**) for ATiO_3_ (A=Li, Na, K, Rb, and Cs).

The reflectivity parameter $R\left(\omega \right)$ illustrates the behavior of photons interacting with the material by describing the proportion of light that reflects from the substance, shown in Figure 7a and obtained from Equation (S12).

Table 4 provides the static values of reflectivity at zero energy, denoted as $R\left(0\right)$, for each of the discussed materials. These findings confirm our previous results on optical features, indicating that the majority of incoming photons are transmitted through these materials in the solar range, despite the rise of $R\left(\omega \right)$ in the UV area. This suggests that light reflection in the solar region is minimal for these compounds. NaTiO_3_ displays the lowest $R\left(\omega \right)$ and, consequently, the highest transmission among all examined perovskite structures, emphasizing its already proposed function in PSCs and Per-SCs. Additionally, the higher reflectivity values for previously identified ferromagnetic compounds, including KTiO_3_, could favor more light trapping in the active layer to increase the efficiency of solar cells. In the UV region, there is a noticeable increase in the reflectivity of all examined perovskite oxides, with NaTiO_3_ exhibiting the highest value. This indicates that a substantial portion of incident light is reflected by these compounds within the UV spectrum.

#### 3.3.6. Energy Loss Function

The energy loss function, L(ω), describes the energy that may be lost by fast electrons during motion [115,122], which is shown in Figure 7b, where L(ω) is calculated using Equation (S13).

Each peak in the L(ω) spectra represents a different plasma frequency, and they all indicate a plasma resonance. The electron energy loss spectra (EELS) of all the examined compounds show several loss peaks in the visible and UV areas, as shown in Figure 7b. The only substance without any peaks in the solar range is the semiconductor compound NaTiO_3_.

Higher energy losses in the UV area of the spectrum are indicated by a rise in L(ω) as a result of increases in photon energy. As listed in Table 4, the most intensive plasmon peak of 1.08, corresponding to the energy of 12 eV, is observed for NaTiO_3_ in the UV region.

The EELS comprises two main segments: the low-loss region, covering energy losses up to approximately 50 eV, and the high-loss zone. Analyzing the valence region (<50 eV) offers insights similar to those obtained from optical spectroscopy, providing valuable information about the materials’ properties and behavior [123]. From the low-loss spectrum, which comprises the zero-loss peak and the plasmon peaks, insights into the band structure and dielectric characteristics of the sample can be derived [123,124]. In line with the previously described optical characteristics, the absence of prominent plasmon peaks in the solar region is evident in the EELS for the semiconductor compound NaTiO_3_, which suggests a clear absence of interaction between this substance and incident light within this specific spectral range. As shown in Figure 7b, the EELS for NaTiO_3_ exhibits a delayed rise occurring after the energy edge of 4 eV and remains close to zero across the majority of the solar region. These estimations validate our earlier proposals that NaTiO_3_ could serve effectively as either a CTL or a bottom TCE in solar cells.

Based on the optical properties of the electronic structures, NaTiO_3_ is considered the preferred candidate among the investigated titanate-based perovskite structures as CTL and TCE in the PSCs and Per-SCs. While no reports exist on the use of NaTiO_3_ as a CTL or TCE, several studies have explored other perovskite oxides with high transparency in the solar spectrum for these functions [30,34,125,126,127]. Our findings indicate the potential suitability of NaTiO_3_ for similar applications, suggesting the need for further experimental validation. Furthermore, optical calculations have confirmed the potential function of ferromagnetic compounds, particularly KTiO_3_, as additional absorbents in the active layer. Although there are no reports on the specific alkali-based titanate perovskite oxides studied here, other metallic and half-metallic compounds have been used successfully as additional absorbents in the active layers of solar cells [37,38,99], which supports the potential application of our investigated compounds.

### 3.4. Elastic and Thermal Properties

Most current research focuses on solving the key problem of device stability rather than increasing efficiency, even though Per-SCs and PSCs have not yet achieved the theoretically projected highest efficiency [128,129]. The endurance of the active layer against moisture and temperature and the structural stability of the solar cell’s components, most notably the ETL, HTL, and electrodes, play a significant role in determining the durability of solar devices [20].

Hence, to attain good mechanical and thermal stability in PSCs and Per-SCs, it is imperative for the CTLs, TCE, and top electrodes to withstand external factors such as elevated pressure and raised temperatures [129]. The elastic characteristics of materials control their behavior under stress, which includes pressure and strain. The elastic constants describe the connection between strain and stress in a material and the bending of a material under stress could be approximated using its elastic constants [130]. Given the importance of stability in the PSC and Per-SC commercialization process, calculations were conducted to determine the elastic constants and derived elastic moduli, including Young’s modulus, bulk and shear moduli, and Poisson’s ratios, for each perovskite oxide, to ascertain their mechanical stability [131,132]. Since elasticity is inherently dynamic, measuring its high and low moduli is more complicated than measuring other characteristics. Similar to rubber-like substances, materials with lower elastic moduli bend easily yet return to their former shape swiftly. Conversely, denser, stiffer materials with larger elastic moduli could withstand larger loads [133,134]. Roof panels for electric cars and folding umbrellas are only two examples of novel uses that might benefit from the incorporation of flexible materials in the construction of PSCs and Per-SCs.

#### Elastic and Thermal Properties of Alkali-Based Titanates

The main isotropic elastic parameters for each perovskite oxide, including the bulk modulus $\left(B_{H}\right)$, shear modulus $\left(G_{H}\right)$, Young’s modulus $\left(E_{H}\right)$, and Poisson’s ratio $\left(\nu_{H}\right)$, obtained from the Hill approximation, are calculated from the elastic constants of the single crystals using Equations (1)–(12) and are summarized in Table 5. Structures with different space groups have different numbers of elastic constants. LiTiO_3_, with its orthorhombic structure and the space group pnma, has nine elastic constants c_11_, c_12_, c_13_, c_22_, c_23_, c_33_, c_44_, c_55_, and c_66_ with calculated values of 77.64, 104.48, 226.00, 76.41, 255.03, and 44.82 GPa, respectively (Table 5). The calculated values of the 13 elastic constants, c_11_, c_12_, c_13_, c_15_, c_22_, c_23_, c_25_, c_33_, c_35_, c_44_, c_46_, c_55_, and c_66_ of the monoclinic structures of NaTiO_3_ in the c2/m space group are also summarized in Table 5. KTiO_3_ and CsTiO_3_, with tetragonal structures in the space group P4mm, have six independent elastic constants, c_11_, c_12_, c_13_, c_33_, c_44,_ and c_66_. Table 5 shows that the elastic constants of CsTiO_3_ are smaller than KTiO_3_. Finally, the trigonal structure of RbTiO_3_, with space group R-3, has eight elastic constants c_11_, c_12_, c_13_, c_14_, c_15_, c_33_, c_44_, and c_66_. The data in Table 5 highlight that across all studied structures, the elastic constant c_11_ consistently emerges as the largest, while the elastic constant with the lowest value varies among the different structures. In order to determine which compound is mechanically and thermally the most stable for each task, the elastic properties of the compounds that are compared are based on the functions they are expected to perform. Thus, we will compare the elastic properties of the metallic compounds LiTiO_3_ and CsTiO_3_, proposed for use as the top metallic electrode (cathode) and, similarly, we will compare the elastic characteristics of ferromagnetic KTiO_3_ and RbTiO_3_, which are suggested as additional absorbents. However, NaTiO_3_ is the only semiconductor structure identified in this study and recommended for use as CTL or TCE and will therefore be evaluated independently based on its elastic properties.

As shown in Table 5, LiTiO_3_ exhibits greater bulk, Young’s, and shear moduli (141.59, 186.99, and 73.05 GPa, respectively), compared to CsTiO_3_ (66.38, 101.18, and 40.61 GPa, respectively). Conversely, KTiO_3_ has higher bulk, Young’s, and shear moduli (137.03, 206.81, and 82.83 GPa, respectively) than RbTiO_3_ (111.11, 91.71, and 33.66 GPa, respectively). Notably, NaTiO_3_ exhibits the smallest values for these moduli (59.28, 79.10, and 30.96 GPa, respectively).

The bulk modulus $\left(B\right)$ and the shear modulus (G) are directly related to the incompressibility of a substance [135,136]. The bulk modulus of a substance is defined by the proportion of direct stress to the corresponding volumetric strain when a structure experiences three equally intense, mutually perpendicular stresses. When a material is subjected to homogeneous pressure throughout its whole surface, the alteration in volume with respect to its initial volume is known as volumetric strain [137]. Thus, evaluating the bulk modulus of a crystal is a particular method of determining its durability, where a high bulk modulus indicates that the structure is resistant to compression [135]. Among the two metallic perovskite structures (LiTiO_3_ and CsTiO_3_), LiTiO_3_ has a larger (B) and is less compressible. Among the ferromagnetic structures (KTiO_3_, RbTiO_3_), KTiO_3_ has the larger (B), whereas the only semiconductor compound, NaTiO_3_, stands out with the lowest (B) value and, as a result, its highest compressibility. The connection between shear stress and shear strain in a material is defined by the shear modulus, which can also be referred to as the modulus of rigidity. As a function of area, shear stress is a tangential force. Despite keeping its volume constant, a body could be deformed by applying tangential stress on one surface while the other surface stays stationary. The substance experiences shear strain due to this distortion, which causes the force-exposed surface to move in the direction of the force [137,138]. As evident from Table 5, LiTiO_3_ and KTiO_3_ have the highest values of shear modulus (G) among the metallic and half-metallic structures, respectively, while the semiconductor compound NaTiO_3_ shows the lowest value and is therefore most easily deformed. The Young’s modulus of elasticity (E) is the stress-to-strain ratio along its longitudinal axis. As a material encounters stress per unit area of its cross-section, the change in length per unit length is known as longitudinal strain, and the force exerted on the substance is known as longitudinal stress [139,140]. The plasticity of a substance can be evaluated by measuring its (E), where a material with a high Young’s modulus is typically regarded as rigid, i.e., not easily bent or compressed [141]. The values of (E) for the investigated structures follow the order of LiTiO_3_ > CsTiO_3_ and KTiO_3_ > RbTiO_3_ for the metallic and ferromagnetic structures, respectively. Once more, the semiconductor compound NaTiO_3_ has the lowest value of (E) at 79.10 (Table 5). Poisson’s ratio $\left(\nu \right)$ is the ratio of lateral strain to longitudinal strain [142], determining the flexibility of a compound where $\left(\nu \right)$ has an optimum value that falls within the range of 0 to 0.5. A smaller $\left(\nu \right)$ indicates greater plastic behavior, while a larger value suggests higher elasticity [136]. The data presented in Table 5 reveal that RbTiO_3_ possesses a $\left(\nu \right)$ value of 0.33, which is higher than that of KTiO_3_ (0.25). LiTiO_3_ has a $\left(\nu \right)$ value of 0.28, just greater than that of CsTiO_3_ (0.25), and the same as NaTiO_3_ with a $\left(\nu \right)$ value of 0.28.

In order to determine the ductility and brittleness of these materials, we have used various elastic parameters, including the Pugh ratio ($(B)/\left(G\right)$) and $\left(\nu \right)$. Ductile materials, unlike brittle ones, undergo deformation under stress without significant alteration in volume [136]. According to Pugh’s criteria, a material is considered to exhibit ductile behavior if the ratio of (B)/$\left(G\right)$ exceeds 1.75. Conversely, if this ratio is less than 1.75, the material is classified as brittle [135]. As shown in Table 5, the Pugh ratio for LiTiO_3_, NaTiO_3_, and RbTiO_3_ are all ≥1.75, categorizing them as ductile materials, with RbTiO_3_ exhibiting the highest value. In contrast, KTiO_3_ and CsTiO_3_ have Pugh ratios of less than 1.75, indicating their brittle characteristics. These findings are further supported by evaluating Poisson’s ratio $\left(\nu \right)$, another important parameter to determine whether a material is ductile or brittle. According to the Frantsevich rule, a substance is brittle if $\left(\nu \right)$ < 0.26 and ductile if $\left(\nu \right)$ > 0.26. The data in Table 5 clearly show that the ductility and brittleness of the compounds derived from Pugh’s ratio are confirmed by their $\left(\nu \right)$ values.

The A factor (2C_44_/(C_11_ − C_12_)) represents the crystal elastic anisotropy factor. When A is equal to 1, it indicates that the substance exhibits elastic isotropic properties. In contrast, in an anisotropic substance, A is not equal to 1 [143]. The estimated A factor for all investigated compounds differs from 1, indicating their anisotropic nature. As shown in Table 5, NaTiO_3_ has the smallest documented A value of 0.16, while RbTiO_3_ has the greatest value of 1.48.

Figure 8 depicts the surface contours representing the spatial variation of Young’s modulus using the Hill scheme for all of the studied crystal structures, demonstrating the anisotropy present in different perovskite materials. Figures S1–S10 further exhibit surface contours illustrating the spatial variation of the bulk modulus and Poisson’s ratio for all structures.

The calculated minimum lattice thermal conductivities (κ_min_) and Debye temperatures ($\theta_{D}$) for each structure are listed in Table 5. There is a close correlation between the $\theta_{D}$ and several physical parameters, such as elastic constants, Debye frequency, specific heat, and melting point [143]. The Debye temperature can be precisely predicted using elastic measurements. Since the Debye temperature in solids is related to interatomic forces, its high value is an indication of powerful bonding in the substance [134]. The data in Table 5 show that all the compounds under investigation display remarkably high Debye temperature values, indicating the presence of robust internal bonding, in the sequence LiTiO_3_ > KTiO_3_ > NaTiO_3_ > RbTiO_3_ > CsTiO_3_, with LiTiO_3_ and KTiO_3_ exhibiting a higher Debye temperature among metallic and half-metallic compounds, respectively.

The thermal endurance of the materials can be assessed by considering their minimum thermal conductivity (κ_min_), which represents the amount of heat conducted through a meter of material thickness for each degree of temperature difference between two sides. Compounds with lower thermal conductivity are generally preferred because they conduct less heat energy [144]. As indicated in Table 5, the minimum lattice thermal conductivity values for LiTiO_3_, NaTiO_3_, KTiO_3_, RbTiO_3_, and CsTiO_3_ are recorded as 0.24, 0.11, 0.12, 0.16, and 0.13 W.m^−1^.K^−1^, respectively. As such, CsTiO_3_ (metallic), NaTiO_3_ (semiconductor), and KTiO_3_ (half-metallic), with their lower minimum thermal conductivities, have the potential to enhance the thermal stability of devices when incorporated into solar cell structures as a cathode, CTL, or TCE, and an additional absorbent in the active layer.

Overall, having considered the electronic and optical characteristics along with the elastic properties of alkali-based titanate perovskite oxides, it is clear that certain compounds could be promising candidates for different functions in solar cells. Integrating the metal CsTiO_3_ compound as a cathodic electrode could significantly enhance the mechanical and thermal stability of the solar cell in comparison to LiTiO_3_. In contrast, NaTiO_3_, the sole semiconductor compound examined in this study, demonstrates favorable elastic properties, suggesting its suitability as a TCE and CTL, thereby potentially enhancing the performance and stability of PSCs and Per-SCs. Moreover, among the ferromagnetic compounds investigated, the utilization of KTiO_3_ as an additional absorber in the active layer also shows substantial potential to improve the mechanical and thermal stability of solar cells.

## 4. Conclusions

We have performed a comprehensive computational study to gain insight into the feasibility of ATiO_3_s perovskites (A=Li, Na, K, Rb, and Cs) as CTLs, TCEs, top metallic electrodes, or additional absorbents in the active layer of polymer/perovskite solar cells. To this end, the structural, optoelectronic, magnetic, thermal, and elastic properties of the ATiO_3_ perovskite oxides in different phases have been investigated using ab initio methods based on the density functional theory.

The calculated tolerance factor confirms the stability of each structure. The density of states and spin-polarized band structure calculations have revealed that LiTiO_3_, KTiO_3_, and RbTiO_3_ have a ferromagnetic nature, while NaTiO_3_ and CsTiO_3_ are non-magnetic structures. The calculated magnetic moments of LiTiO_3_, KTiO_3_, and RbTiO_3_ are 0.836, 0.781, and 0.820 µB per formula unit, respectively, and the O(2p) states are the main source of the spin magnetic moment in LiTiO_3_, KTiO_3_, and RbTiO_3_. We found that KTiO_3_ and RbTiO_3_ show similar electronic properties as half-metallic ferromagnetic materials, while LiTiO_3_ and CsTiO_3_ are metallic. Metallic compounds may be good candidates as top electrodes, whereas ferromagnetic materials could also be applied as alternative absorbents in the active layer of PSCs and Per-SCs.

NaTiO_3_ shows semiconductor behavior with a direct band gap of 2.771 eV. The PDOS results of the compounds reveal that O(2p) orbitals occupy the VB, whereas the contribution of Ti(3d) orbitals is prominent in the CBM. Based on its electronic structures, NaTiO_3_, with a band gap larger than 2, is considered a primary candidate to employ as CTL and transparent conductive bottom electrodes of PSCs and Per-SCs.

We have also calculated various optical parameters, including the refractive index, extinction coefficient, reflectivity, energy loss function, adsorption coefficient, and optical conductivity. The electronic structure, along with optical analyses, show that NaTiO_3_ is the best candidate for CTLs and bottom TCEs in solar cells, considering their low optical conductivity and absorptivity, minimal refractive index and reflectivity of the visible light, the wider band gap, and high transparency in the solar region. KTiO_3_, with higher values of these parameters, is a promising ferromagnetic compound to be employed as an additional absorbent in the active layer of PSCs and Per-SCs.

Important elastic and thermal parameters, including the bulk modulus, shear modulus, Young’s modulus, and Poisson’s ratio, as well as minimum lattice thermal conductivities, were also calculated to evaluate the mechanical and thermal stability of the compounds. NaTiO_3_ with lower elastic constants and minimum thermal conductivity is again a good candidate for CTLs and bottom TCE to achieve a more flexible device with enhanced thermal and mechanical stability. Moreover, the lower values calculated for the elastic constants and thermal conductivity of the KTiO_3_ compound, consistent with its optical characteristics, support its significant potential for use in solar cells to enhance their efficiency and optical and thermal stability. At the same time, the same optical and elastic calculations revealed that CsTiO_3_, compared to other metallic compounds, is a valid candidate to be applied as the top electrode and an active layer absorbent to improve mechanical flexibility and thermal stability.

## Figures and Tables

**Figure 8:**
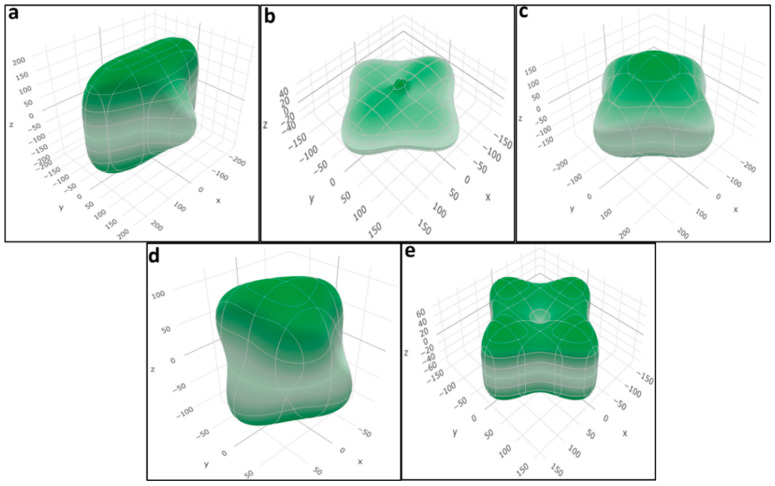
Calculated surface contours of spatial dependence of Young’s modulus (in GPa) obtained from Hill approximation of the (**a**) LiTiO_3_, (**b**) NaTiO_3_, (**c**) KTiO_3_, (**d**) RbTiO_3_, and (**e**) CsTiO_3_ extracted data for elastic calculations.

**Table 1:** Crystal structure, space group, volume, lattice parameters (a, b, c, α, β, and γ), formation energy, and t-factor of various perovskite bulk crystal structures.

Bulk Unit Cell Formula	Crystal Structures (Space Group)	Volume (Å^3^)	a (Å)	b (Å)	c (Å)	α (°)	β (°)	γ (°)	ΔH_f_ [77,78] (eV/Atom)	t-Factor
(LiTiO_3_)_4_	Orthorhombic (Pnma)	199.587	5.550	7.001	5.136	90.00	90.00	90.00	−2.454	0.962
(NaTiO_3_)_4_	Monoclinic (C2/m)	135.444	5.647	5.647	5.492	100.67	100.67	122.97	−2.510	0.855
KTiO_3_	Tetragonal (P4mm)	62.569	3.969	3.969	3.972	90.00	90.00	90.00	−2.443	1.000
(RbTiO_3_)_2_	Trigonal (R-3)	160.963	6.966	6.966	6.966	46.25	46.25	46.25	−2.386	1.041
CsTiO_3_	Tetragonal (P4mm)	76.328	3.901	3.901	5.016	90.00	90.00	90.00	−2.307	1.005

**Table 2:** Calculated partial and total spin magnetic moments per formula unit of perovskite oxides ATiO_3_ (A=Li, Na, K, Rb, and Cs).

	m_A_ (µB)	m_Ti_ (µB)	m_3O_ (µB)	m_tot_ (µB/f.u.)
A=Li	−0.004	−0.062	0.902	0.836
A=Na	0	0	0	0
A=K	−0.012	−0.164	0.957	0.781
A=Rb	0.006	−0.1	0.914	0.820
A=Cs	0	0	0	0

**Table 3:** The spin-up and spin-down E_VBM_, E_CBM_, and E_g_ in eV from the TDOS of perovskite oxides ATiO_3_ (A=Li, Na, K, Rb, Cs).

	E_g_ Spin-Up	E_VBM_ Spin-Up	E_CBM_ Spin-Up	E_g_ Spin-Down	E_VBM_ Spin-Down	E_CBM_ Spin-Down	Electronic Nature
LiTiO_3_	0	0.063	2.317	0	1.005	2.283	metallic
NaTiO_3_	2.771	−0.021	2.750	2.771	−0.021	2.750	semiconductor
KTiO_3_	2.333	−0.090	2.243	0	0.687	2.095	half-metallic
RbTiO_3_	3.177	−0.122	3.055	0	0.547	2.964	half-metallic
CsTiO_3_	0	0.410	2.456	0	0.410	2.456	metallic

**Table 4:** Values of dielectric constant, $\varepsilon_{1}(0)$, refractive index, n(0), and reflectance, R(0), at zero energy and maximum values of absorption coefficient, $\alpha_{max}(\omega )$, optical conductivity, $\sigma_{max}(\omega )$, and energy loss function, $L_{max}(\omega )$.

Name	$\varepsilon_{1}(0)$	n(0)	R(0)	$\alpha_{max}(\omega )$ (10^4^/cm)	$\sigma_{max}(\omega )$ (10^16^/s)	$L_{max}(\omega )$
LiTiO_3_	22.68	4.76	0.43	123.53	15.88	0.58
NaTiO_3_	3.14	1.77	0.08	188.80	25.76	1.08
KTiO_3_	31.34	5.60	0.49	100.63	12.29	0.70
RbTiO_3_	10.60	3.26	0.28	148.60	21.66	0.45
CsTiO_3_	10.51	3.24	0.28	143.03	17.74	0.50

**Table 5:** Calculated elastic constants (c_ij_, in GPa), bulk modulus (in GPa), Young’s modulus (in GPa), shear modulus (in GPa), Poisson’s ratio, minimum lattice thermal conductivity (in W.m^−1^.K^−1^), and Debye temperature (in K) obtained from Hill approximation of monoclinic ATiO_3_ (A=Li, Na, K, Rb, and Cs) perovskites.

Elastic Parameters	LiTiO_3_	NaTiO_3_	KTiO_3_	RbTiO_3_	CsTiO_3_
c_11_	287.71	205.61	310.72	149.23	210.18
c_12_	77.64	61.32	76.09	95.27	77.44
c_13_	104.48	18.79	67.85	85.52	45.29
c_14_	⸺	⸺	⸺	0.533	⸺
c_15_	⸺	0.40	⸺	-6.49	⸺
c_22_	226.00	199.69	⸺	⸺	⸺
c_23_	76.41	22.81	⸺	⸺	⸺
c_25_	⸺	0.93	⸺	⸺	⸺
c_33_	255.03	51.94	210.30	169.40	43.86
c_35_	⸺	2.25		⸺	⸺
c_44_	60.61	11.52	72.27	40.05	50.17
c_46_	⸺	2.06	⸺	⸺	⸺
c_55_	107.38	16.98	⸺	⸺	⸺
c_66_	44.82	47.61	71.92	26.98	35.50
Bulk modulus (B_H_)	141.59	59.28	137.03	111.11	66.38
Young’s modulus (E_H_)	186.99	79.10	206.81	91.71	101.18
Shear modulus (G_H_)	73.05	30.96	82.83	33.66	40.61
Poisson’s ratio (ν_H_)	0.28	0.28	0.25	0.36	0.25
Pugh’s ratio	1.94	1.91	1.65	3.30	1.63
Cauchy’s pressure	17.03	49.80	4.17	55.22	27.27
Anisotropy factor	0.57	0.16	0.61	1.48	0.75
Minimum thermal conductivity (κ_min_)	0.24	0.11	0.12	0.16	0.13
Debye temperature (θ_D_)	711.90	453.40	684.50	398.30	380.40

## Data Availability

The raw data supporting the conclusions of this article will be made available by the authors on request due to privacy.

## Supplementary Materials

The following supporting information can be downloaded at: https://www.mdpi.com/article/10.3390/molecules29143355/s1, Figure S1. Calculated surface contours of spatial dependence of shear modulus (in GPa) obtained from Hill approximation of LiTiO_3_; Figure S2. Calculated surface contours of spatial dependence of Poisson’s ratio (in GPa) obtained from Hill approximation of LiTiO_3_; Figure S3. Calculated surface contours of spatial dependence of shear modulus (in GPa) obtained from Hill approximation of NaTiO_3_; Figure S4. Calculated surface contours of spatial dependence of Poisson’s ratio (in GPa) obtained from Hill approximation of NaTiO_3_; Figure S5. Calculated surface contours of spatial dependence of shear modulus (in GPa) obtained from Hill approximation of KTiO_3_; Figure S6. Calculated surface contours of spatial dependence of Poisson’s ratio (in GPa) obtained from Hill approximation of KTiO_3_; Figure S7. Calculated surface contours of spatial dependence of shear modulus (in GPa) obtained from Hill approximation of RbTiO_3_; Figure S8. Calculated surface contours of spatial dependence of Poisson’s ratio (in GPa) obtained from Hill approximation of RbTiO_3_; Figure S9. Calculated surface contours of spatial dependence of shear modulus (in GPa) obtained from Hill approximation of CsTiO_3_; Figure S10. Calculated surface contours of spatial dependence of Poisson’s ratio (in GPa) obtained from Hill approximation of CsTiO_3_.
