# Supplementary material for: A Density Functional Theory Study of the Physico-Chemical Properties of Alkali Metal Titanate Perovskites for Solar Cell Applications
Source: Molecules. 2024 Jul 17;29(14):3355. doi: 10.3390/molecules29143355 (PMC11279520; doi:10.3390/molecules29143355)
Supplement: Supplementary file 1 [file molecules-29-03355-s001.zip › molecules-2981553-supplementary.pdf]

Supporting Information

A Density Functional Theory Study of the Physico-Chemical Properties of Alkali Metal Titanate Perovskites for Solar Cell Applications

Shirzad Jouybar [1], Leila Naji [1,*], Saeedeh Sarabadani Tafreshi [1,2,*], Nora H. de Leeuw [2,3,*]

[1] Department of Chemistry, AmirKabir University of Technology, No. 350, Hafez Avenue, Valiasr Square, Tehran 1591634311, Iran

[2] School of Chemistry, University of Leeds, Leeds LS2 9JT, UK

[3] Department of Earth Sciences, Utrecht University, 3584 CB Utrecht, The Netherlands

* Correspondence: leilanaji@aut.ac.ir (L.N.); s.s.tafreshi@aut.ac.ir or s.sarabadanitafreshi@leeds.ac.uk (S.S.T.); n.h.deleeuw@leeds.ac.uk (N.H.d.L.)

$$B_V = \frac{1}{9}(C_{11} + C_{22} + C_{33}) + \frac{2}{9}(C_{12} + C_{13} + C_{23})$$ Equation (S1)

$$B_R = \frac{1}{(S_{11} + S_{22} + S_{33}) + 2(S_{12} + S_{13} + S_{23})}$$ Equation (S2)

$$G_V = \frac{1}{15}(C_{11} + C_{22} + C_{33} - C_{12} - C_{13} - C_{23}) + \frac{1}{5}(C_{44} + C_{55} + C_{66})$$ Equation (S3)

$$G_R = \frac{1}{4(S_{11} + S_{22} + S_{33}) - 4(S_{12} + S_{13} + S_{23}) + 3(S_{44} + S_{55} + S_{66})}$$ Equation (S4)

$$E_H = \frac{9B_H G_H}{3B_H + G_H}$$ Equation (S5)

$$\nu_H = \frac{3B_H - 2G_H}{2(3B_H + G_H)}$$ Equation (S6)

$$\kappa_{min} = \left\{\frac{1}{3}\left[2\left(\frac{1}{2+2\nu_H}\right)^{-3/2} + \left(\frac{1}{3-6\nu_H} + \frac{2}{3+3\nu_H}\right)^{-3/2}\right]\right\}^{-1/3} k_B N_A^{2/3} \frac{n^{2/3}\rho^{1/6}E_H{}^{1/2}}{M^{2/3}}$$ Equation (S7)

$$n(\omega) = \left[\frac{\varepsilon_1(\omega)}{2} + \sqrt{\frac{\varepsilon_1^2(\omega) + \varepsilon_2^2(\omega)}{2}}\right]^{\frac{1}{2}}$$ Equation (S8)

$$\mathrm{k}(\omega) = \left[\frac{-\varepsilon_1(\omega)}{2} + \sqrt{\frac{\varepsilon_1^2(\omega) + \varepsilon_2^2(\omega)}{2}}\right]^{\frac{1}{2}}$$ Equation (S9)

$$\alpha(\omega) = 2\omega\mathrm{k}(\omega) = 2\omega\left[\frac{-\varepsilon_1(\omega)}{2} + \sqrt{\frac{\varepsilon_1^2(\omega) + \varepsilon_2^2(\omega)}{2}}\right]^{\frac{1}{2}}$$ Equation (S10)

$$\sigma(\omega) = {\alpha(\omega)n(\omega)c}/{4\pi}$$ Equation (S11)

$$R(\omega) = \frac{(n(\omega)-1)^2 + k(\omega)^2}{(n(\omega)+1)^2 + k(\omega)^2} = \left|\frac{\sqrt{\varepsilon}-1}{\sqrt{\varepsilon+1}}\right|^2$$ Equation (S12)

$$L(\omega) = \frac{\varepsilon_2(\omega)}{\varepsilon_1^2(\omega) + \varepsilon_2^2(\omega)}$$ Equation (S13)

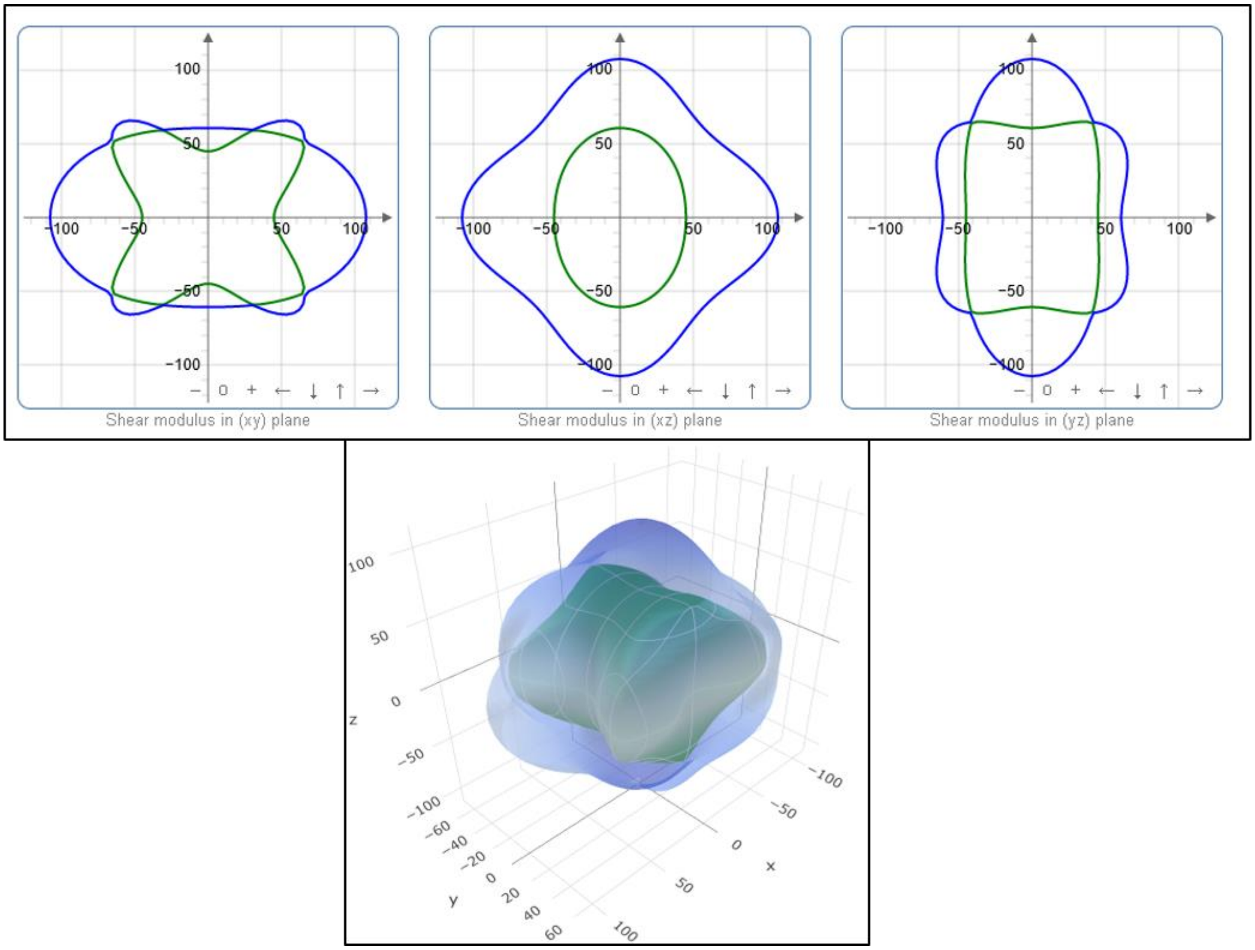

Figure S1. Calculated Surface contours of spatial dependence of shear modulus (in GPa) obtained from Hill approximation of $LiTiO_3$.

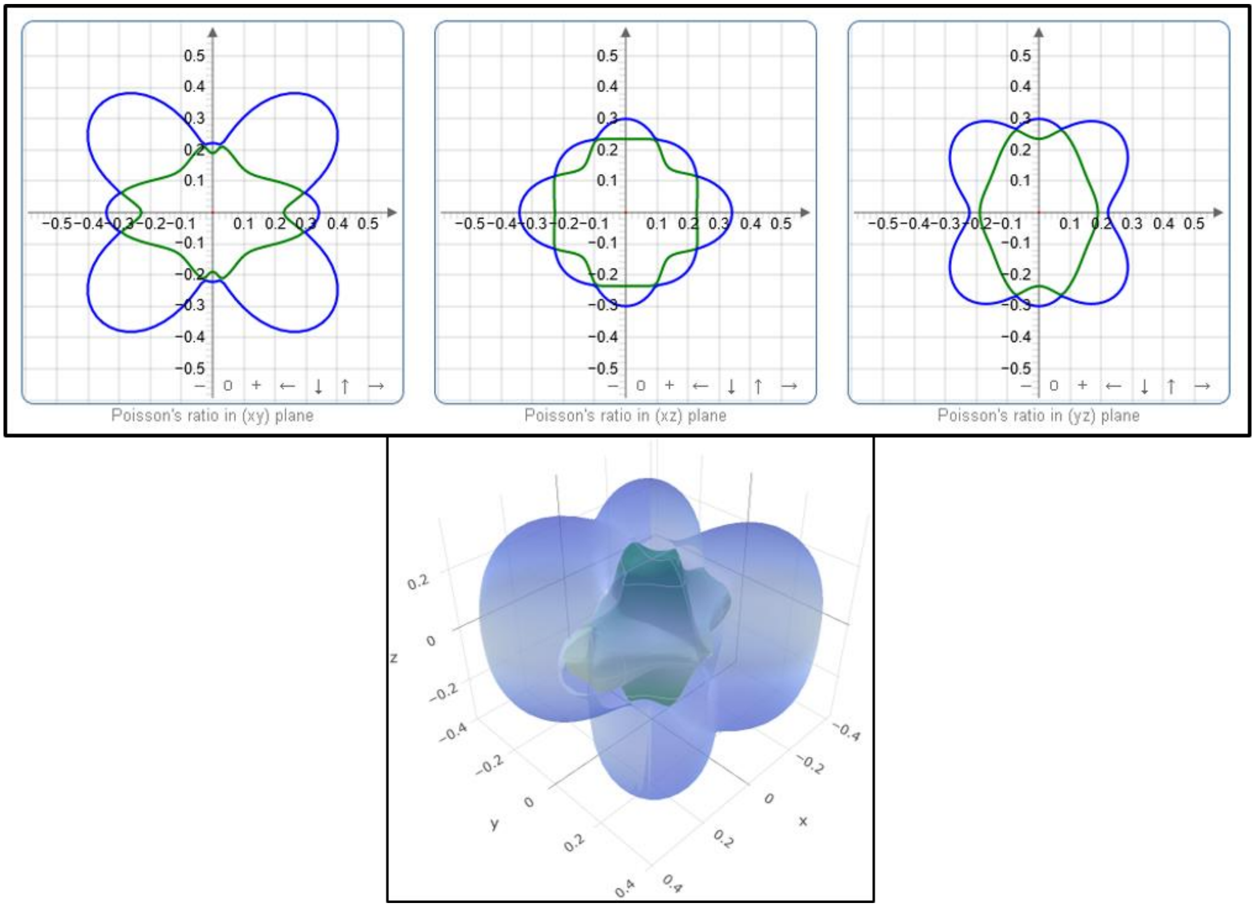

Figure S2. Calculated Surface contours of spatial dependence of Poisson's ratio (in GPa) obtained from Hill approximation of $LiTiO_3$.

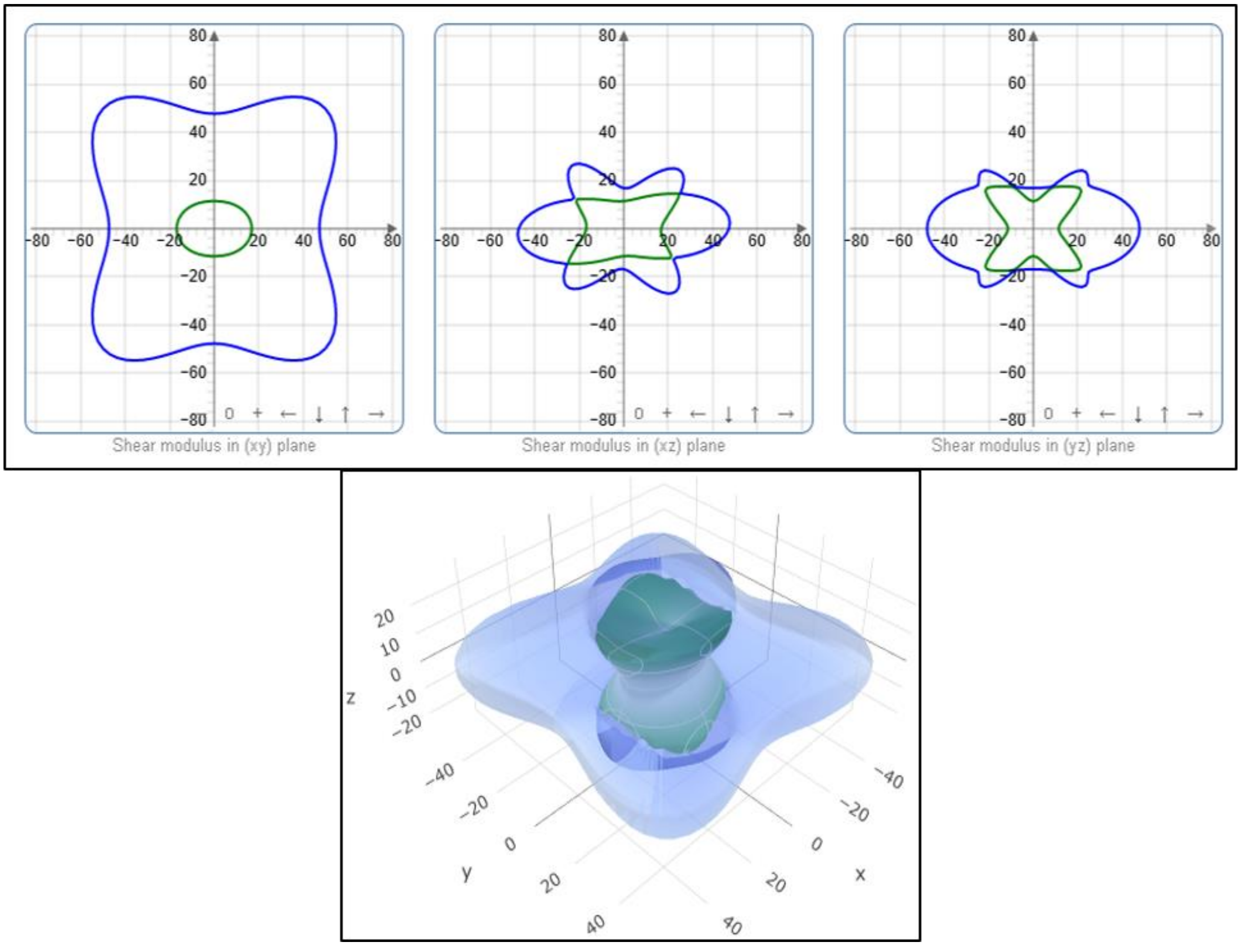

Figure S3. Calculated Surface contours of spatial dependence of shear modulus (in GPa) obtained from Hill approximation of $NaTiO_3$.

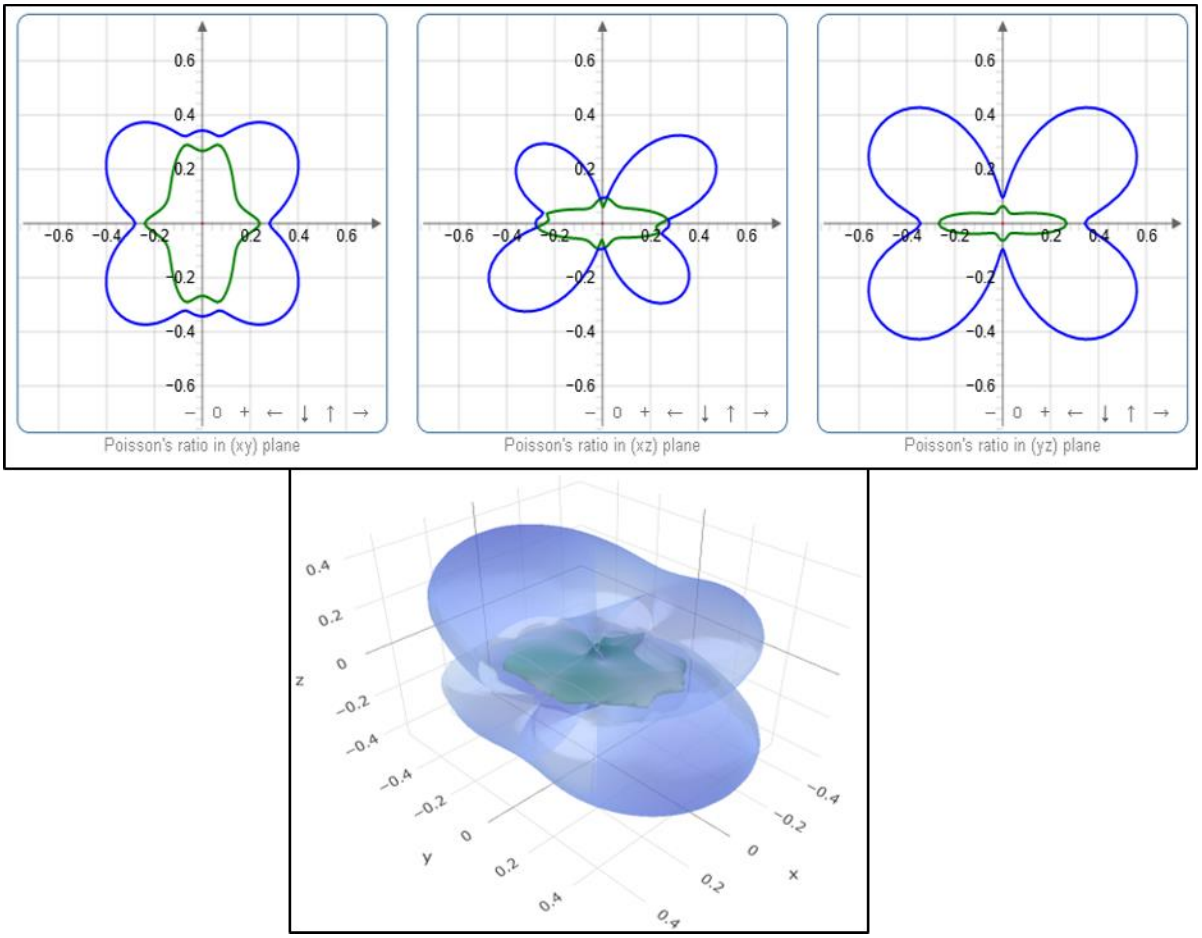

Figure S4. Calculated Surface contours of spatial dependence of Poisson's ratio (in GPa) obtained from Hill approximation of $NaTiO_3$.

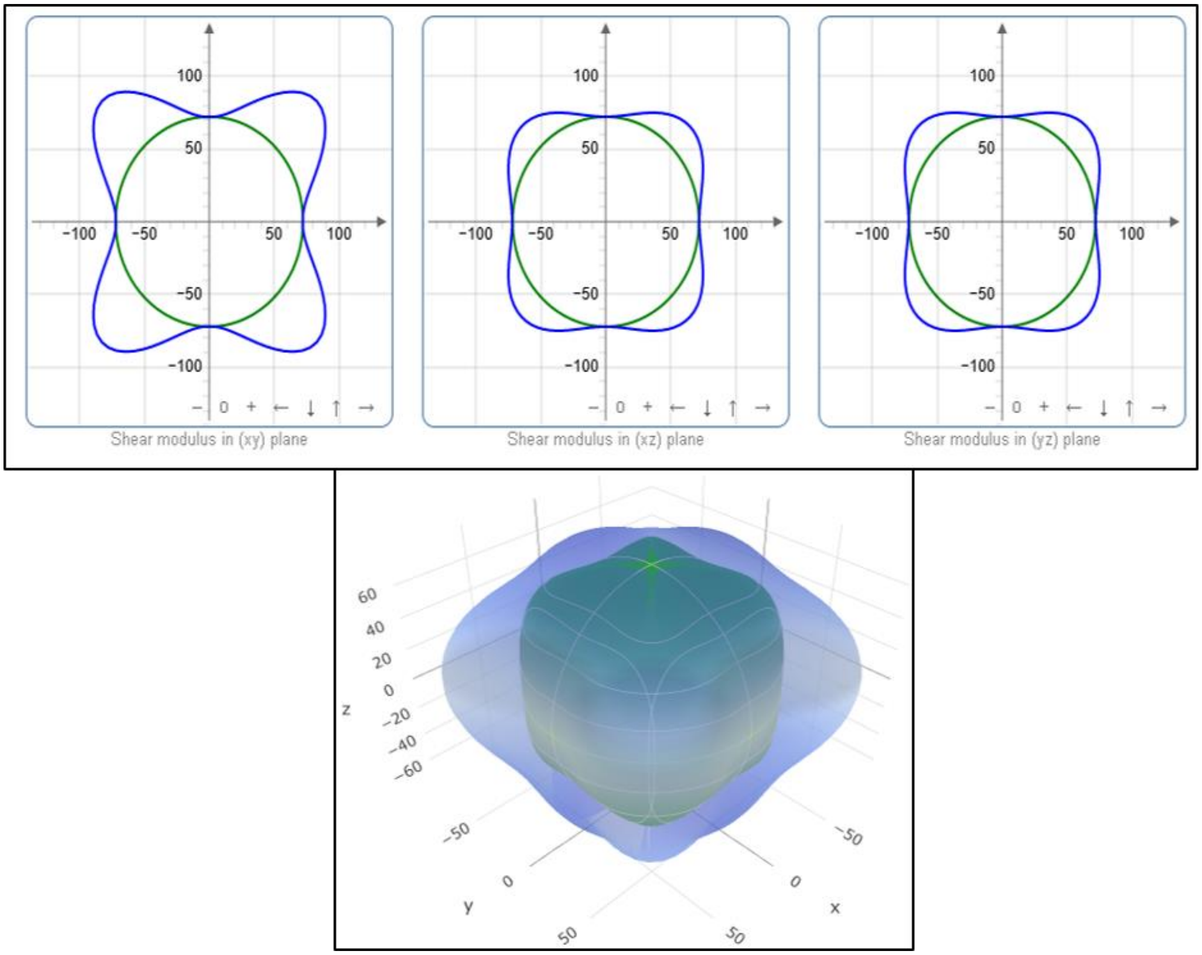

Figure S5. Calculated Surface contours of spatial dependence of shear modulus (in GPa) obtained from Hill approximation of $KTiO_3$.

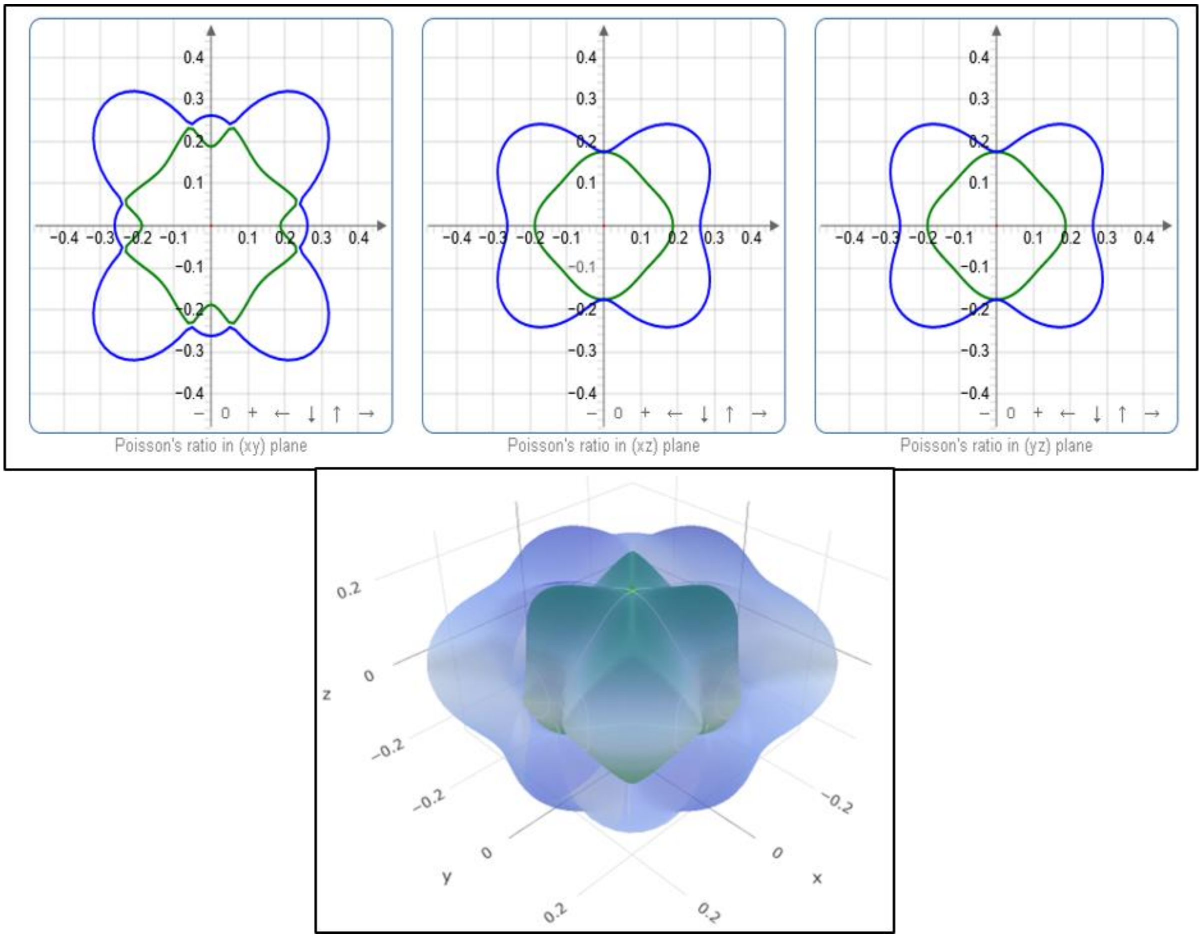

Figure S6. Calculated Surface contours of spatial dependence of Poisson's ratio (in GPa) obtained from Hill approximation of $KTiO_3$.

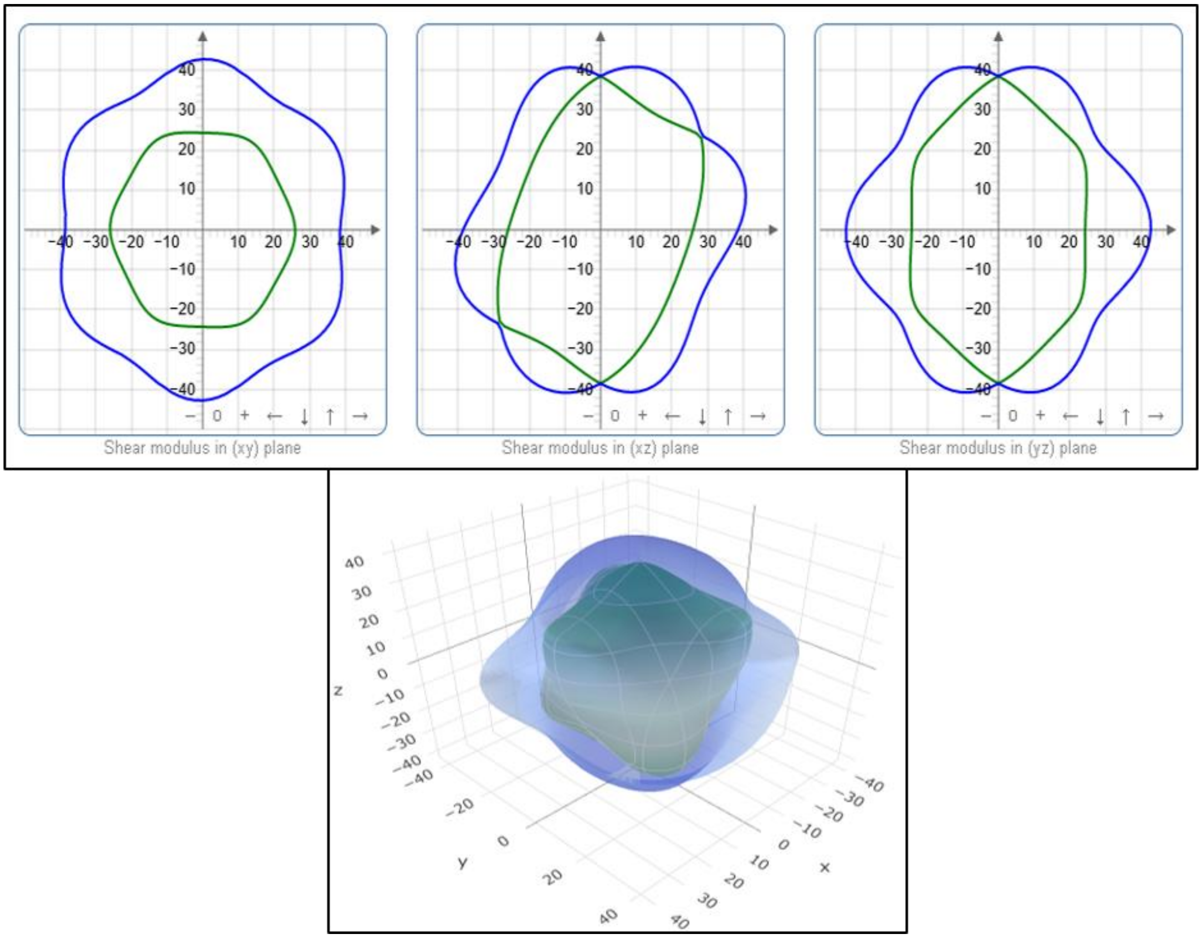

Figure S7. Calculated Surface contours of spatial dependence of shear modulus (in GPa) obtained from Hill approximation of $RbTiO_3$.

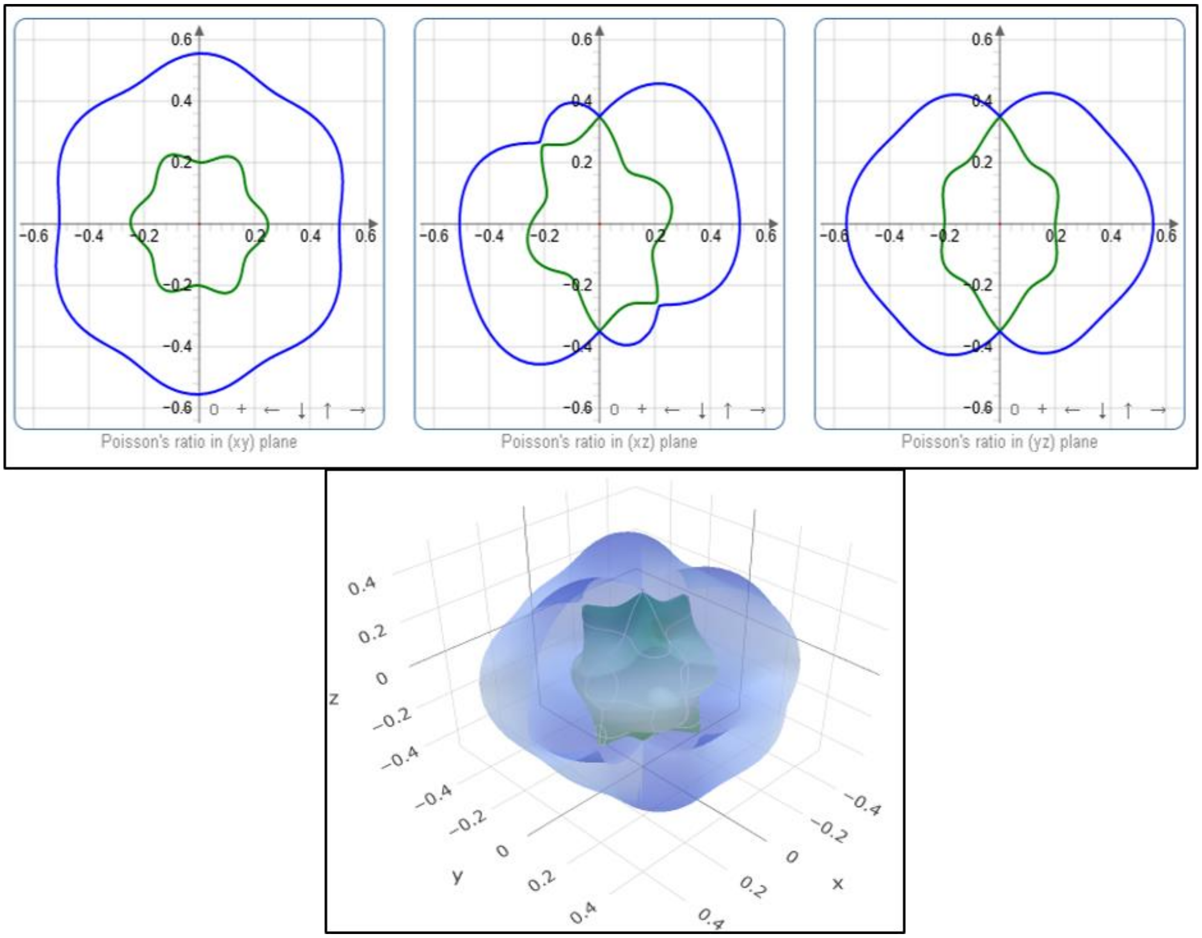

Figure S8. Calculated Surface contours of spatial dependence of Poisson's ratio (in GPa) obtained from Hill approximation of $RbTiO_3$.

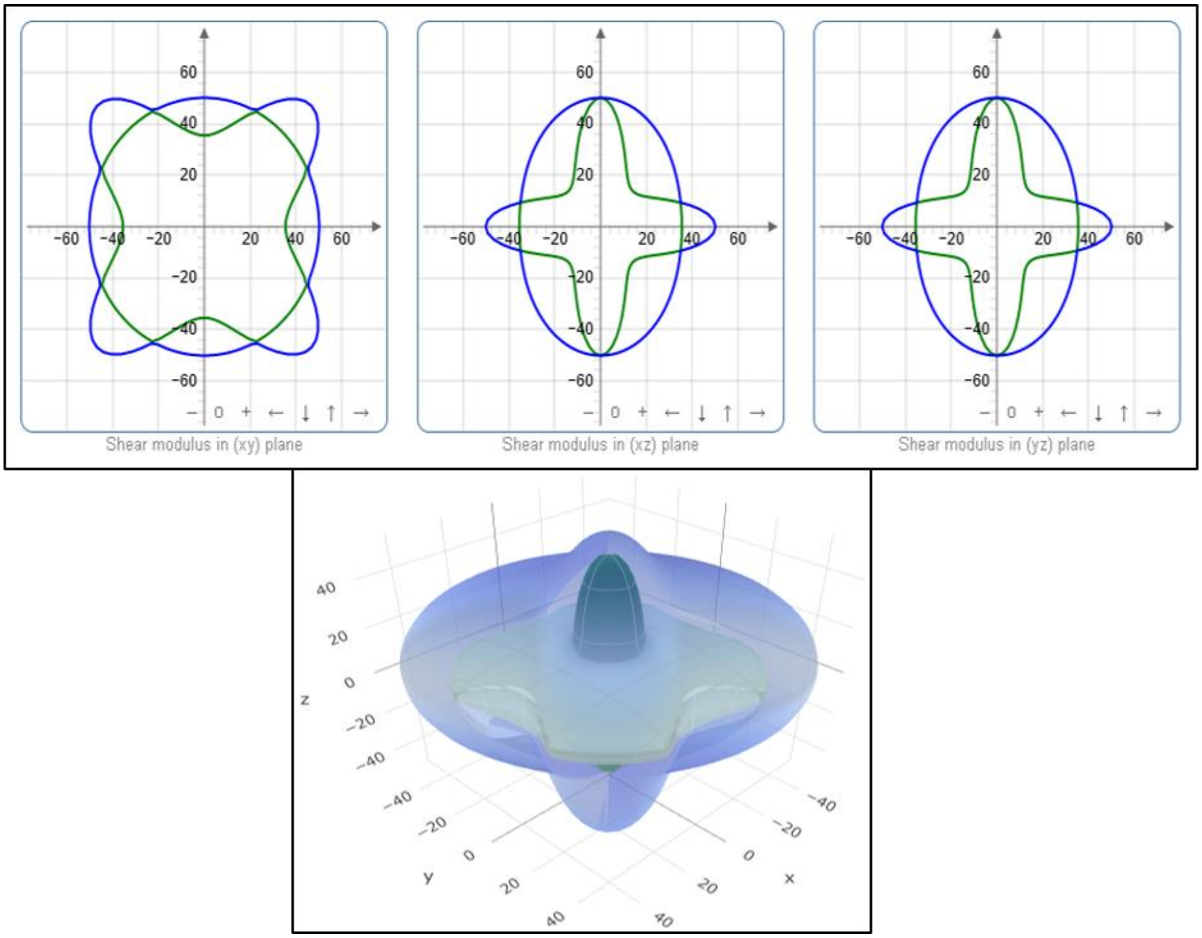

Figure S9. Calculated Surface contours of spatial dependence of shear modulus (in GPa) obtained from Hill approximation of $CsTiO_3$.

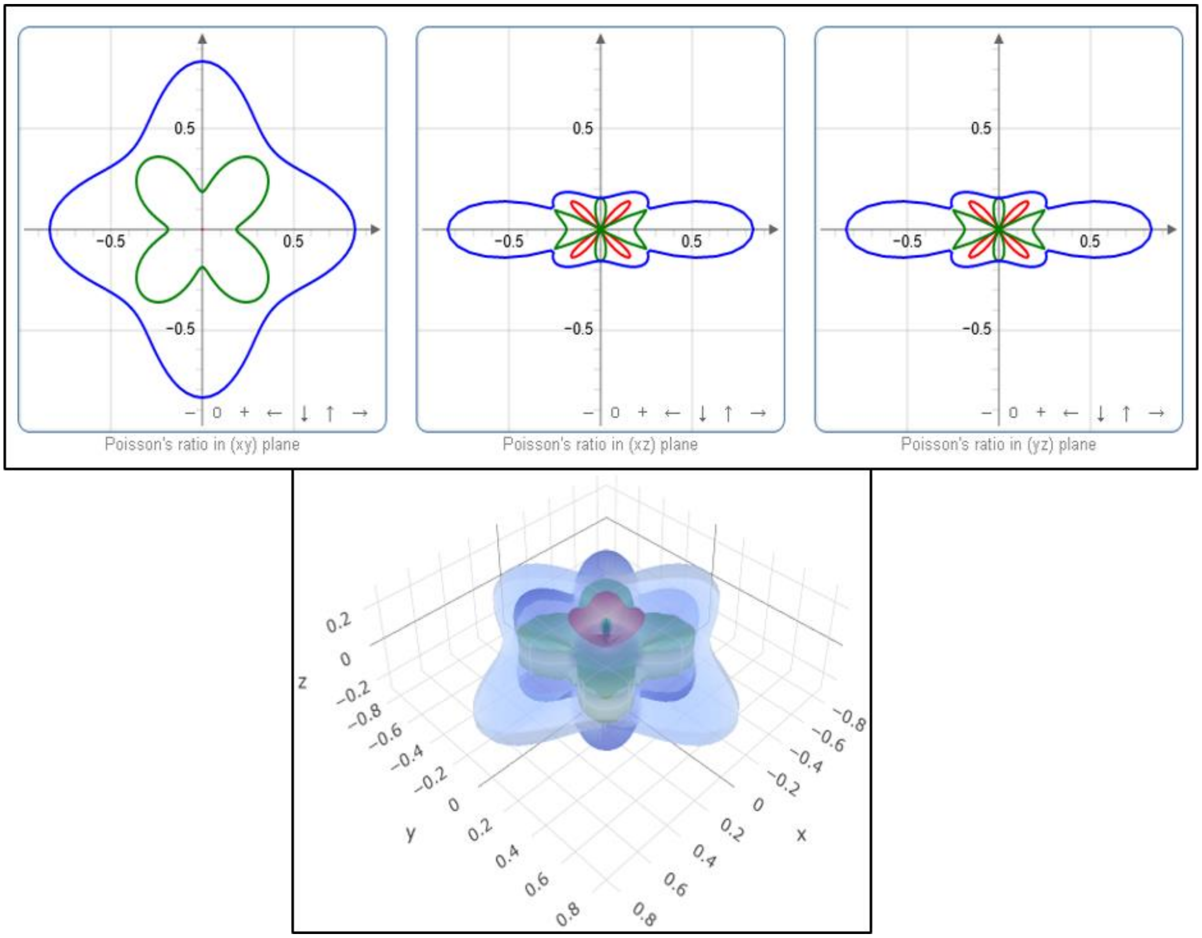

Figure S10. Calculated Surface contours of spatial dependence of Poisson's ratio (in GPa) obtained from Hill approximation of $CsTiO_3$.
